# Development of a transcription factor-based biosensor strain for reporting α-terpineol production *via* the alcohol-dependent hemiterpene pathway in *Escherichia coli*

**DOI:** 10.1039/d5cb00310e

**Published:** 2026-02-18

**Authors:** Catherine A. Odhiambo, Isaac A. Ali, Gavin J. Williams

**Affiliations:** a Department of Chemistry, NC State University Raleigh North Carolina 27695 USA gjwillia@ncsu.edu; b Comparative Medicine Institute, NC State University Raleigh North Carolina 27695 USA

## Abstract

Terpenes constitute a vast and industrially important class of natural products. Yet, microbial production of many high-value terpenoids remains limited by the difficulty of rationally engineering their biosynthetic pathways and the lack of high-throughput screening systems that directly report product formation. This challenge is especially acute for monoterpene alcohols such as α-terpineol (1), whose biosynthesis in heterologous hosts requires coordinated precursor formation, cyclization, and water-capture chemistry. Here, we develop a transcription factor–based whole-cell biosensor strain capable of detecting 1 by engineering the *p*-cumate repressor CymR through structure-guided directed evolution. Guided by a model of the putative ligand-binding pocket, focused libraries at residues implicated in effector accommodation yielded variants with dramatically improved sensitivity. This culminated in the CymR variant 3-A8, which exhibits a 22-fold increase in dynamic range relative to wild-type. Using this optimized biosensor, we demonstrate *in vivo* monitoring of 1 production in *E. coli* by coupling it to an artificial alcohol-dependent hemiterpene (ADH) pathway and downstream modules expressing GPPS and α-terpineol synthase. The integrated biosensor–production system effectively distinguishes the complete biosynthetic pathway from deletions and reports intracellular titers consistent with GC-MS quantification. Together, these results provide the first biosensor for monocyclic monoterpene alcohols and establish a compact, modular framework for high-throughput screening and pathway optimization. This platform sets the stage for accelerating the discovery, engineering, and scalable bioproduction of valuable isoprenoids and other terpene-derived natural products.

## Abstract 2

Development of a genetically-encoded biosensor enabled reporting production of α-terpineol in an engineered *E. coli* strain.

## Graphical abstract

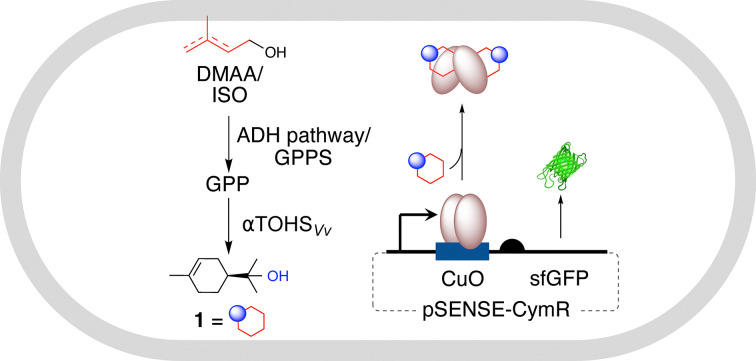

## Introduction

**Fig. 1:**
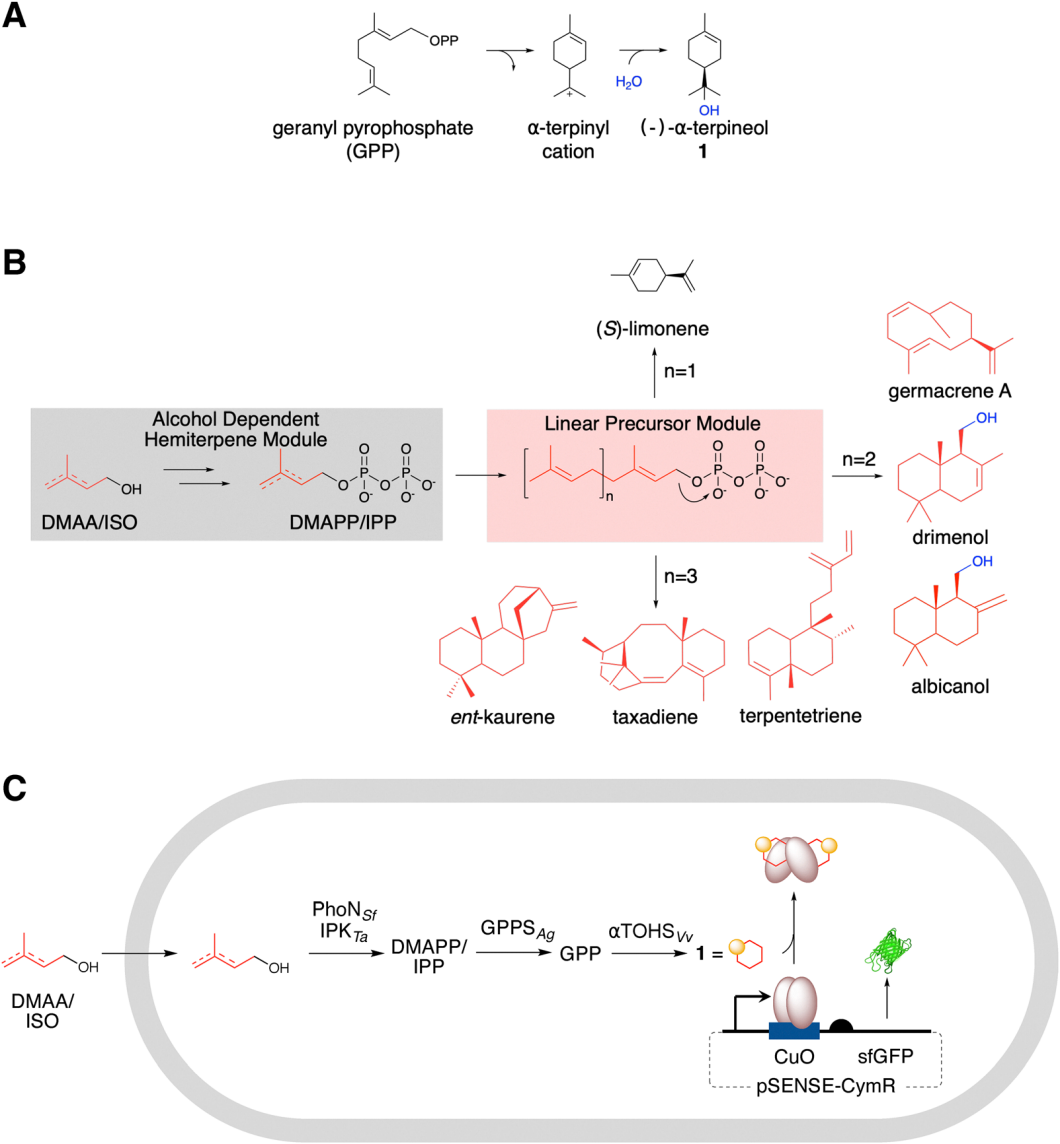
Engineered biosynthesis of monoterpenes. (A) Biosynthesis of 1*via* α-terpineol synthase, showing hydroxylation *via* water capture from the terpinyl cation. (B) Overview of terpene biosynthesis *via* allylic-stabilized diphosphates accessed *in situ via* the artificial pathway. (C) This study: alcohol-dependent hemiterpene pathway for 1 production and detection *via* a transcription-factor-based biosensor.

Methods are highly sought after for the scalable, efficient biosynthetic production of isoprenoids.^1–7^ Their extensive chemical diversity supports a wide range of biological functions and industrial application.^8^ Monoterpenes consist of two isoprene units (C10) and constitute the main components of flavor and fragrance in essential oils.^9–11^ Many well-known, canonical, monocyclic monoterpenes are oxygenated and exhibit a wide range of biological activities, including antimicrobial, antifungal, anti-inflammatory, antiviral, antioxidant, and antitumor properties.^12,13^ These attributes make oxygenated monocyclic terpenes attractive for the development of novel drugs.^12^ For instance, known for its lilac-like aroma, α-terpineol 1 (Fig. 1A) has broad industrial applications in perfumes and cosmetics.^14^ Large-scale commercial production of 1 relies on chemical synthesis *via* α-pinene hydration and isomerization.^15,16^ This process typically requires harsh acidic conditions (hydrochloric, sulfuric, and phosphoric acids), and yields are low.^17^ Therefore, there is significant interest in developing microbial strains capable of producing 1, which offers a promising alternative to chemical synthesis.^18^ The biosynthesis of 1 in yeast from the 10-carbon precursor geranyl pyrophosphate proceeds *via* the condensation of dimethylallyl pyrophosphate (DMAPP) and isopentenyl pyrophosphate (IPP) *via* geranyl diphosphate synthase from *Abies grandis* (GPPS_Ag_), which then undergoes cyclization by α-terpineol synthase from grapevine species *Vitis vinifera* (αTOHS_Vs_, Fig. 1A).^18^ This reaction produces a terpinyl cation, which is subsequently stabilized through the capture of H_2_O, forming 1. Enzymes that catalyze water capture–mediated hydroxylation reactions represent a potential alternative to cytochrome P450 monooxygenases for introducing hydroxyl groups into terpene scaffolds.^19^ The spontaneous or enzyme-assisted hydration of carbocation intermediates avoids the dependence on heme cofactors and NAD(P)H required by P450s and could target sites inaccessible to conventional P450s. This mechanism has been described for several terpene synthases, thereby expanding the known chemical diversity of terpene biosynthesis.^19–24^ Yet, multiple bottlenecks in the pathway have been identified, making rational design-based optimization challenging and high-throughput approaches highly attractive.^25,26^

We and others have developed an artificial alcohol-dependent hemiterpene (ADH) pathway for terpene biosynthesis that can be coupled to downstream steps in heterologous host systems.^27–29^ The ADH pathway includes PhoN from *Shigella flexneri* (PhoN_Sf_) and isopentenyl phosphate kinase from *Thermoplasma acidophilum* (IPK_Ta_). Together, PhoN and IPK catalyze the sequential phosphorylation of exogenously supplied dimethylallyl alcohol (DMAA) and isopentenol (ISO) to yield DMAPP and IPP.^30,31^ Recently, the phosphorylation activity of PhoN_Sf_ was improved by mutagenesis.^29^ Some cyclic isoprenoids have been produced using the ADH pathway (Fig. 1B),^24,32,33^ in addition to alkylated tryptophan derivatives,^31^ but not terpene alcohols. Therefore, developing the biosynthesis of 1 offers an opportunity to expand the scope of ADH pathways and to assess their capacity for producing water-captured hydroxylated terpenes. We anticipated that two additional expression modules could be combined with the ADH pathway to generate 1 in *Escherichia coli* (*E. coli*) (Fig. 1C). The linear precursor module expresses the geranyl diphosphate synthase GPPs_Ag_, which catalyzes the condensation of DMAPP and IPP to form GPP. The third module, dedicated to cyclization, expresses αTOHS_Vv_, which cyclizes GPP to produce 1.^34,35^ However, further optimization of the ADH pathway or downstream steps is likely necessary to improve terpene titers. Given the challenges associated with rational redesign, improving 1-titers and other terpene outputs is likely to be more effective through high-throughput screening of enzyme and pathway variants.

Transcription factor-based biosensors provide an opportunity to develop a high-throughput approach for rapidly identifying and overcoming unknown bottlenecks in 1 production. Unlike GC-MS assays,^36^ transcription factor biosensors enable screening of up to 10^8^ mutants per library and are highly customizable.^37^ Traditionally, transcription factor-based biosensors convert small molecule binding into the expression of reporter proteins such as green fluorescent protein (GFP).^38^ Many reliable transcription factor-analyte pairs have been developed,^39–41^ including MyrR for the detection of acyclic monoterpenes and CamR for the biosensor-based measurement of selected bicyclic (camphene, borneol, eucalyptol, and fenchol) monoterpenes.^42,43^ However, none have yet been specifically developed that respond to monocyclic terpene alcohols or report the activity of terpene alcohol synthases. We aimed to develop a biosensor for 1 detection that enables high-throughput strain screening. We chose the *p*-cumate repressor (CymR) from the *p*-cymene catabolism operon of *Pseudomonas putida F*1^44^ as a starting point for developing a 1 biosensor (Fig. 1C). This system has been effectively used to study gene expression in various organisms in response to *p*-cumate.^45^ Given the structural similarities between cumate and certain monocyclic isoprenoids, it is plausible that engineered CymR variants could exhibit a response to 1 and/or related monoterpenes. Additionally, transcription factors often respond to effectors beyond their native ligands, making them attractive scaffolds for directed evolution.^38,46–52^ This provided a rationale for exploring CymR as a transcription factor scaffold for developing a biosensor capable of reporting 1 biosynthesis.

An AlphaFold computational model^53^ facilitated docking^54–56^ of 1 and the identification of the putative effector binding site. We set out to test whether a 1-biosensor could be developed by focusing mutagenesis on the transcription factor's putative binding pocket, thereby minimizing library sizes and the effort needed to screen them. Capitalizing on the simplicity of the prototype ADH pathway, which requires only two downstream enzymes to access 1, we utilized the biosensor to distinguish the productivity of a panel of variant strains. This approach, utilizing biosensor-guided assays of ADH-supported terpene production strains, holds potential for application to various high-value terpenes. It is expected to contribute to the design-build-test-learn cycle by providing a deeper understanding of design constraints as we develop terpene biosensors and engineered biosynthetic pathways.

## Experimental

### Bacterial strains, plasmids, and materials

Synthetic oligonucleotides were purchased from Integrated DNA Technologies (Coralville, IA, USA). The CymR fragment was purchased from Twist Bioscience (San Francisco, CA). Enzymes used for DNA manipulation and plasmid isolation *via* a mini-prep kit were purchased from New England Biolabs (Ipswich, MA, USA). Polymerase chain reactions were conducted with Q5 Hot Start High Fidelity 2× master mix. All plasmids were verified by DNA sequencing of either the target gene (Azenta, Research Triangle Park, NC, USA) or the full-length plasmid (Plasmidsaurus, Louisville, KY, USA).

The *E. coli* DH5α and TOP10 strains (Invitrogen) were used for standard cloning procedures and maintenance of genetic constructs. Recombinant enzyme expression and pathway reconstitution were performed in BL21(DE3) cells. Unless stated otherwise, the enzymes used in this work were handled according to the manufacturer's recommendations. The cells were heat-shocked at 42 °C for 45 s and recovered in 1 mL Super Optical broth with Catabolite repression (SOC) medium (MP Biomedicals). Bacteria were grown in Luria-Bertani (Fisher Scientific) supplemented with ampicillin and streptomycin (Sigma Aldrich) as appropriate. Optical Density at 600 nm (OD_600_) was measured on a CaryBio® UV-visible spectrophotometer. Unless otherwise stated, all solids were dissolved in 18.2 mΩ resistance H_2_O from Barnstead water. A 10× liquid PBS from Fisher Scientific was used to prepare a final (1×) solution for the cell pellet resuspension, and 96-deepwell microplates were purchased from Fisher Scientific. Chemical compounds (1–9) and dimethyl sulfoxide (DMSO) used for the CymR biosensor characterization assay were purchased from Sigma-Aldrich (St. Louis, MO, USA). Each monoterpenoid was prepared in DMSO at a stock concentration of 10–400 mM. Dimethylallyl alcohol (DMAA) and isopentenol (ISO), used for the biosynthesis of 1, were purchased from Fisher Scientific. Absorbance and fluorescence readings were taken in clear flat-bottom and black flat-bottom 96-well microplates (Greiner Bio-One), respectively, using a BioTek Hybrid Synergy 4 plate reader. Ethyl acetate was used to extract the target biosynthesis compound 1. All other reagents were reagent grade or better.

### Homology modeling of CymR and docking studies

The structure of 1 was optimized for docking using the default MM2 energy minimization parameters in ChemDraw 3D. This was saved in mol 2 format, then converted to pdbqt in Open Babel.^57^ The CymR homology model was prepared for docking in AutoDock tools.^54–56^ All the water molecules were removed before generating a pdbqt file. The ligand and receptor were docked using AutoDock Vina. Docking results were generated for a grid box of 64 × 50 × 40, grid spacing of 1.00, and exhaustiveness = 20, using the center coordinates of 32 × −8 × 44, the grid box center coordinates to ensure that the entire receptor, CymR model, was encompassed within the grid box.

### General procedure for microplate screening of CymR variants

Single colonies from mutant libraries in *E. coli* BL21 (DE3), along with relevant wild-type or parent strains, were picked from LB agar plates supplemented with ampicillin (100 µg mL^−1^) and used to inoculate 500 µL of LB media containing ampicillin (100 µg mL^−1^) in deep 96-well microplates. The cultures were covered by Aeraseal and incubated for 16 h at 37 °C with shaking at 350 rpm. Volumes of 5 µL from the 16 h culture were transferred to each corresponding well, which contained 490 µL of LB media with 100 µg mL^−1^ ampicillin, covered with Aeraseal, and incubated for 5 h at 37 °C with shaking at 350 rpm. Of the two microplates, one was induced by adding 5 µL of compound 1 to a final concentration of 0.5 mM, and the other received 5 µL of DMSO, a negative control. The two plates were covered with a ThermalSeal wrap and incubated for an additional 16 h at 37 °C with shaking at 350 rpm. The cultures were centrifuged at 1509 × *g* for 10 min, after which the cell pellet was resuspended in 600 µL of 1× phosphate-buffered saline (PBS). Two 100 µL of each cell suspension was transferred into a transparent Greiner 96-well microplate and a Greiner 96-well black microplate. The optical density of the cells was analyzed at 600 nm using the transparent microplate, and the fluorescence was measured at 485 nm excitation and 510 nm emission using the black microplate. The fluorescence was divided by the OD_600_ to yield a normalized GFP fluorescence value.

### Dose–response analysis of wild-type and variant CymR biosensor strains

Single colonies of the wild-type or variant CymR biosensor strains in *E. coli* BL21(DE3) were picked from LB agar plates and inoculated into 25 mL of LB containing 100 µg mL^−1^ ampicillin. Cultures were incubated at 37 °C with shaking at 350 rpm until OD_600_ reached 0.8. Then, 495 µL of culture was transferred into the deep 96-well plate and induced with equal volumes (5 µL) of the effectors (1–9) at concentrations from 0.1 to 4.0 mM into the appropriate wells. For controls, 5 µL of DMSO was added to three as added to wells corresponding to each CymR variant. The plates were covered with ThermalSeal wrap and incubated for 16 h at 37 °C with shaking at 350 rpm. The cells were harvested by centrifugation at 1509 × *g* and 4 °C for 10 min, then resuspended in 600 µL of 1× phosphate-buffered saline (PBS), pH 7.5. A 100 µL aliquot of the cell suspension was transferred to clear and black flat-bottom 96-well plates for OD_600_ and fluorescence analysis (ex 485 nm/em 510 nm), respectively. Unless noted otherwise, dose–response data points represent the averages of triplicate measurements, with error bars indicating the standard deviation. Normalized GFP fluorescence values were calculated by dividing fluorescence intensity by the corresponding OD_600_ values. The normalized GFP expression without the effector (GFP_0_) was subtracted from each normalized fluorescence in the presence of the effector. Effector concentrations and normalized fluorescence intensities were plotted and analyzed using the Hill equation in GraphPad Prism 10.
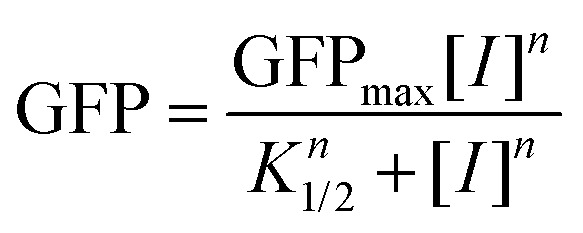
here, GFP_max_ is the maximum normalized GFP expression, [*I*] is the effector concentration, *n* is the Hill coefficient that quantifies the cooperativity of the protein, and *K*_1/2_ is the effector concentration at half-maximal normalized fluorescence.

### *In vivo* production of isoprenoid precursors for biosynthesis and detection of 1

Single colonies of the two-plasmid biosensor/production strain *E. coli* BL21(DE3) pSENSE-3-A8 + pCDF-PhoN_Sf_/IPK_Ta_/GPPS_Ag_/αTOHS_Vv_ or various deletions were picked from LB agar plates and used to inoculate 25 mL of LB 1 : 1 ratio of streptomycin and ampicillin at a concentration of 50 µg mL^−1^ with shaking at 250 rpm for 5 h (OD_600_ ∼0.8–1.0). At this point, IPTG was added to each culture tube at a final concentration of 0.5 mM. Then, 495 µL of IPTG-induced culture was transferred into a deep 96-well plate, and 5 µL of a DMAA/ISO mixture (800 and 400 mM, respectively) was added to the *E. coli* strain. The plates were covered with ThermalSeal wrap and incubated for 20 h at 28 °C with shaking at 350 rpm. For GC-MS analysis, a 250 µL sample was transferred into 1.7 mL Eppendorf tubes, and 125 µL of ethyl acetate was added to each tube. The tube was centrifuged at 4816 × *g* for 10 min at 25 °C. The organic layer was extracted and transferred into the glass tubes for GC-MS analysis. GC-MS analysis was conducted on an Agilent 8860 using a J and W CycloSil-B, with a 30 m × 250 µm × 0.25 µm capillary column. Helium (99.99% purity) was used as a carrier gas at a flow rate of 1.1 mL min^−1^. The oven was programmed to start at 50 °C for 3 min, then increase at 25 °C min^−1^ to 100 °C, followed by a 10 °C min^−1^ increase to 140 °C, and finally a 20 °C min^−1^ increase to 250 °C. The injection port was set at 250 °C on pulsed splitless mode. The injection volume was 1 µL. The EI mode 70 eV. The mass data were acquired after a 4.2 min solvent delay in scan mode, ranging from 30 to 500 *m*/*z*.

The remaining cells were harvested by centrifugation at 1509 × *g* and 4 °C for 10 min, then resuspended in 125 µL of 1× phosphate-buffered saline (PBS), pH 7.5. A 100 µL aliquot of the cell suspension was transferred to clear and black flat-bottom 96-well plates for OD_600_ and fluorescence analysis (ex 485 nm/em 510 nm), respectively. Normalized GFP fluorescence values were calculated by dividing fluorescence intensity by the corresponding OD_600_ values. Concentrations and relative fluorescence intensities were plotted and analyzed *via* the Hill equation with GraphPad Prism 10.

## Results and discussion

### Detection of 1*via* structure-guided mutagenesis of CymR effector binding-pocket

**Fig. 2:**
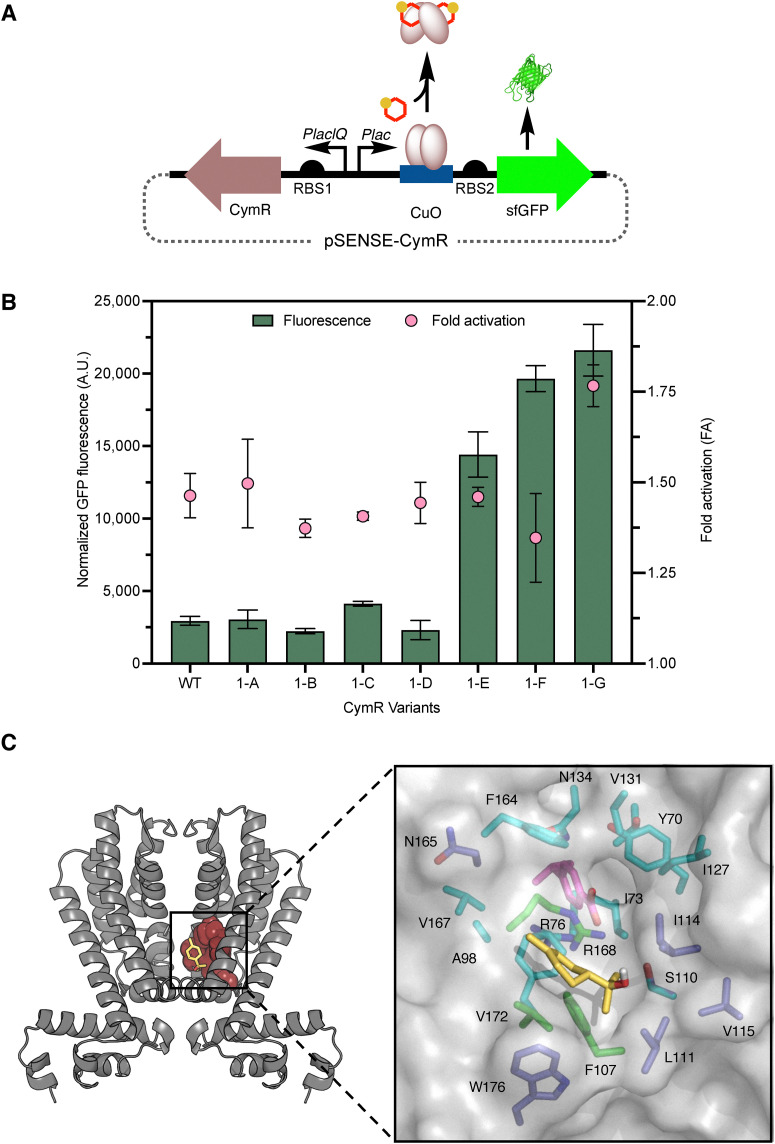
Performance of wild-type and the first-generation CymR variants with 1. (A) Synthetic genetic circuit CymR regulatory system. (B) The fluorescence signal output for the wild-type and variant biosensor strains at 4.0 mM 1. Fold activation is the ratio of normalized fluorescence in the presence (4.0 mM) and absence of 1. (C) CymR homology model with the putative 1 (yellow sticks) binding site (blue sticks) and cumate (purple sticks) binding site (cyan sticks). Sites targeted for engineering are shown as green sticks.

To establish an *E. coli* platform for detecting 1, we constructed an artificial repressor genetic circuit based on our previously described pSENSE platform.^46,51,58^ It features two constitutive promoters (*P*_lacIQ_ and *P*_lac_) that regulate the expression of CymR and superfolder GFP (*sf*GFP), respectively (Fig. 2A an Table S1, and Fig. S1, SI).^51^ On the same platform, RBS1 is positioned upstream of the *CymR* gene and downstream of *P*_lacIQ_, while RBS2 is located upstream of the *sfGFP* gene and downstream of *P*_lac_ (Fig. 2A). The performance characteristics of the wild-type CymR biosensor strain were first assessed by determining its fluorescence response to varying concentrations (0.2–4.0 mM, in the range of typical product titers) of 1 fed into *E. coli* strains that housed the wild-type biosensor plasmid (Fig. S2, SI). The fold-activation of the biosensor strain was also determined by determining the ratio of fluorescence output of the biosensor strain in the presence of 1 (4.0 mM) and its absence (Fig. 2B and Table 1). As anticipated, given the lack of aromatic ring compared to cumate, and differences in size, hydrophobicity, and polarity (Fig. S3, SI), the fluorescence response of the wild-type biosensor with 1 was very low across the entire effector concentration range tested, providing a dynamic range of only 2900 ± 300 and a fold-activation of only 1.5 ± 0.4 (Fig. 2B and Table 1). To be more suitable for reporting 1-production in engineered *E. coli* strains, the poor fluorescence output and fold-activation of the biosensor strain need to be improved.

**Table 1:** Performance features of wild-type and mutant CymR biosensor strains

CymR variants	CymR mutations	GFP_4.0 mM_^a^	*K*_1/2_ b (mM)	*n* c	Fold activation^d^
WT	Wild type	2900 ± 300	4.5 ± 0.8	2.3 ± 0.6	1.5 ± 0.06
1-A	F107A	3050 ± 600	N.D.^e^	1.4 ± 0.4	1.5 ± 0.1
1-B	R168L	2200 ± 200	N.D.^e^	1.2 ± 0.3	1.4 ± 0.03
1-C	V172A	4100 ± 200	5.5 ± 3.6	1.3 ± 0.2	1.4 ± 0.01
1-D	F107A/R168L	14 400 ± 1600	N.D.^e^	1.3 ± 0.4	1.4 ± 0.06
1-E	F107A/V172A	19 700 ± 900	N.D.^e^	1.6 ± 0.2	1.5 ± 0.03
1-F	R168L/V172A	2300 ± 700	N.D.^e^	1.0 ± 0.5	1.3 ± 0.1
1-G	F107A/R168L/V172A	21 600 ± 2000	N.D.^e^	2.0 ± 0.4	1.8 ± 0.06
2-C11	R168E/V172G	14 600 ± 400	3.7 ± 1.2	2.0 ± 0.3	1.6 ± 0.08
2-B11	R168E/V172S	25 200 ± 900	2.5 ± 0.5	1.4 ± 0.1	2.1 ± 0.04
2-E11	R168Q/V172S	17 100 ± 400	N.D.^e^	1.4 ± 0.2	2.0 ± 0.02
2-F2	F107G/R168Q/V172T	35 000 ± 1000	4.3 ± 1.3	2.1 ± 0.3	2.5 ± 0.1
3-A8	F107S/R168E/V172S	43 000 ± 800	3.6 ± 1.0	1.2 ± 0.1	3.5 ± 0.05

a Normalized fluorescence at 4.0 mM of 1. Fluorescence at 0 mM 1 (corrected by OD_600_) subtracted from the 4.0 mM 1 (corrected by OD_600_).

b Concentration of the effector at half maximum relative GFP fluorescence.

c Hill coefficient, a measure of cooperativity within the CymR biosensor. Values > 1 indicate positive cooperativity.

d Ratio of normalized GFP fluorescence (+) **1**/(−) **1**.

e Not detected.

To rationally identify CymR mutations that affect the inducer response, a model of CymR was generated using AlphaFold, based on the recent crystal structure of a putative transcriptional regulator from *Pseudomonas aeruginosa PAO*1.^59–61^ This was analyzed alongside docking studies to guide the selection of residues within 4 Å of the putative 1-binding pocket for targeted mutagenesis (Fig. 2C). This analysis identified three key residues for mutagenesis: Phe107, which π-stacks against 1; Arg168, which likely functions as a gatekeeper; and Val172, which likely contributes to the hydrophobic stabilization of the binding pocket.^62^ Notably, docking suggested that the native effector, *p*-cumate, binds more deeply into the CymR ligand binding pocket than 1. To improve the de-repression of CymR by 1, we hypothesized that: (1) replacing Arg168 with a smaller residue could open the putative effector entrance; (2) substituting Phe107 with a smaller residue could enlarge the binding pocket to accommodate 1 better; and (3) replacing Val172 with a smaller residue could further improve access to 1. A total of seven CymR mutants, including three single mutants, three double mutants, and one triple mutant, were designed to test these hypotheses: F107A (denoted 1-A); R168L (1-B); V172A (1-C); R168L/V172A (1-D); F107A/R168L (1-E); F107A/V172A (1-F); and F107A/R168L/V172A (1-G). Each targeted residue was substituted with Ala or Leu to reduce the side chain volume while retaining hydrophobicity to potentially interact with 1. The performance characteristics of the CymR variant strains were evaluated by measuring their fluorescence response to varying concentrations (0.2–4.0 mM) of 1 fed into *E. coli* strains containing the mutant biosensor plasmids (Fig. S2, SI). Dynamic range values were derived from the normalized fluorescence at 4.0 mM 1, as the response curves did not fully plateau, preventing reliable fitting of the Hill function. The fold-activation for each biosensor strain was determined at 4.0 mM 1 (Table 1). The fold activations for the single and double mutants were comparable to the wild-type CymR, ranging from 1.4 to 1.5 (Fig. 2B and Table 1). However, despite the leaky *sf*GFP expression of 1-E and 1-F, both biosensor strains also exhibited ∼5-fold higher fluorescence signal output in the presence of 1 (Fig. 2B). Among all tested variants, the triple mutant 1-G outperformed the single and double mutants, achieving a dynamic range of 21 600 ± 2000 and a fold activation of 1.8 ± 0.06 (Fig. 2B and Table 1). The enhanced performance of the triple mutant supports the accuracy of the model-generated putative effector binding pocket (Fig. 2C). This, in turn, aligns with our hypothesis that enlarging the effector binding pocket facilitates 1-binding and de-repression. However, the fold-activation and dynamic range likely need further improvement to be useful for screening 1-production.

**Fig. 3:**
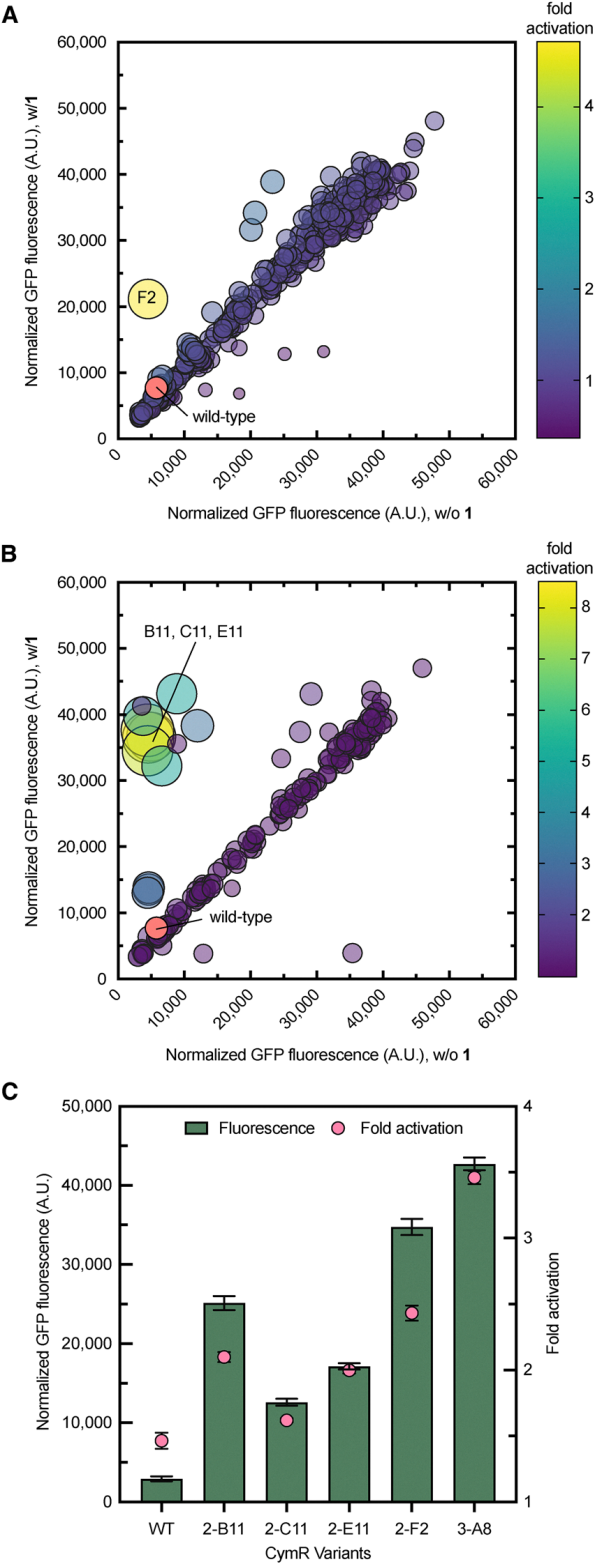
Performance of wild-type and second-generation CymR variants with 1. (A) Responses of individual members of the triple site library (F107X/R168X/V172X) with 1 (0.5 mM) and without 1. The relative size of the bubble corresponds to the fold activation of the library member with respect to the wild-type strain. Variant F2 (yellow bubble) was selected for further analysis. (B) Site-saturation mutagenesis library distribution of the double site library (R168X/V172X). Variants B11, C11, and E11 (yellow bubbles) were selected for further analysis. (C) GFP fluorescence of the wild-type and variant biosensor strains with 4.0 mM of 1, normalized to the culture density. Error bars represent the standard error of the mean (*n* = 3).

Given the impact of mutations at Phe107, Arg168, and Val172 on enhancing the biosensor response with 1, we sought to investigate whether multisite saturation mutagenesis at these residues could further enhance the biosensor's response to 1 while minimizing the screening burden, which is usually associated with large libraries. To do this, we aimed to balance sequence diversity with practical feasibility by creating focused libraries that probe the most informative part of the sequence without covering every possibility. This was defined by positions previously associated with ligand recognition. Two complementary libraries were designed: one targeting the simultaneous randomization of all three residues (F107X/R168X/V172X) and another focusing on the two sites closest to the predicted 1-binding pocket (R168X/V172X). A total of ∼600 and ∼200 library members from the triple and double saturation libraries, respectively, were screened for fluorescence in the presence (0.5 mM) and absence of 1 in microplates (Fig. 3A and B). Library members with at least 2-fold higher fold-activation than the wild-type biosensor were chosen for further analysis, including 2-F2 (F107G/R168Q/V172T), from the triple site library, 2-B11 (R168E/V172S), 2-C11 (R168E/V172G), and 2-E11 (R168Q/V172S) from the double site library. Next, the biosensor strains were evaluated by quantifying GFP fluorescence at various concentrations of 1 (Fig. 3C and Table 1). Notably, the 2-F2 dynamic range (35 000, RFU) was 14-fold higher than that of the wild type strain (2500, RFU) (Fig. 3C and Table 1). Under the same conditions as tested above, the dynamic range of the double site saturation variants, 2-B11, 2-C11, and 2-E11, was 6-, 3-, and 4-fold higher than that of the wild-type, respectively (Fig. 3C). Variant 2-F2 showed a half-maximal activation constant (*K*_1/2_) of 4.3 ± 1.3, which was slightly lower than that of 2-C11 (3.7 ± 1.2) (Table 1). Notably, 2-B11 demonstrated approximately a 2-fold improvement in sensitivity compared to 2-F2. Overall, these results identify 2-B11 as the most promising variant for the second round of iterative engineering.

Most prominently, screening only a small fraction of the theoretical variants in the three-site (∼8%) and two-site (∼50%) saturation libraries was sufficient to identify variants with a significantly improved dynamic range and *K*_1/2_ with 1. The ability to screen a larger portion of the two-site library could have facilitated the discovery of more confirmed hits than from the three-site library. Alternatively, targeting the two residues closest to the effector binding site could have been more efficient.

### Effects of mutagenesis at Phe107 on the performance of the CymR double mutant R168E/V172S (2-B11)

**Fig. 4:**
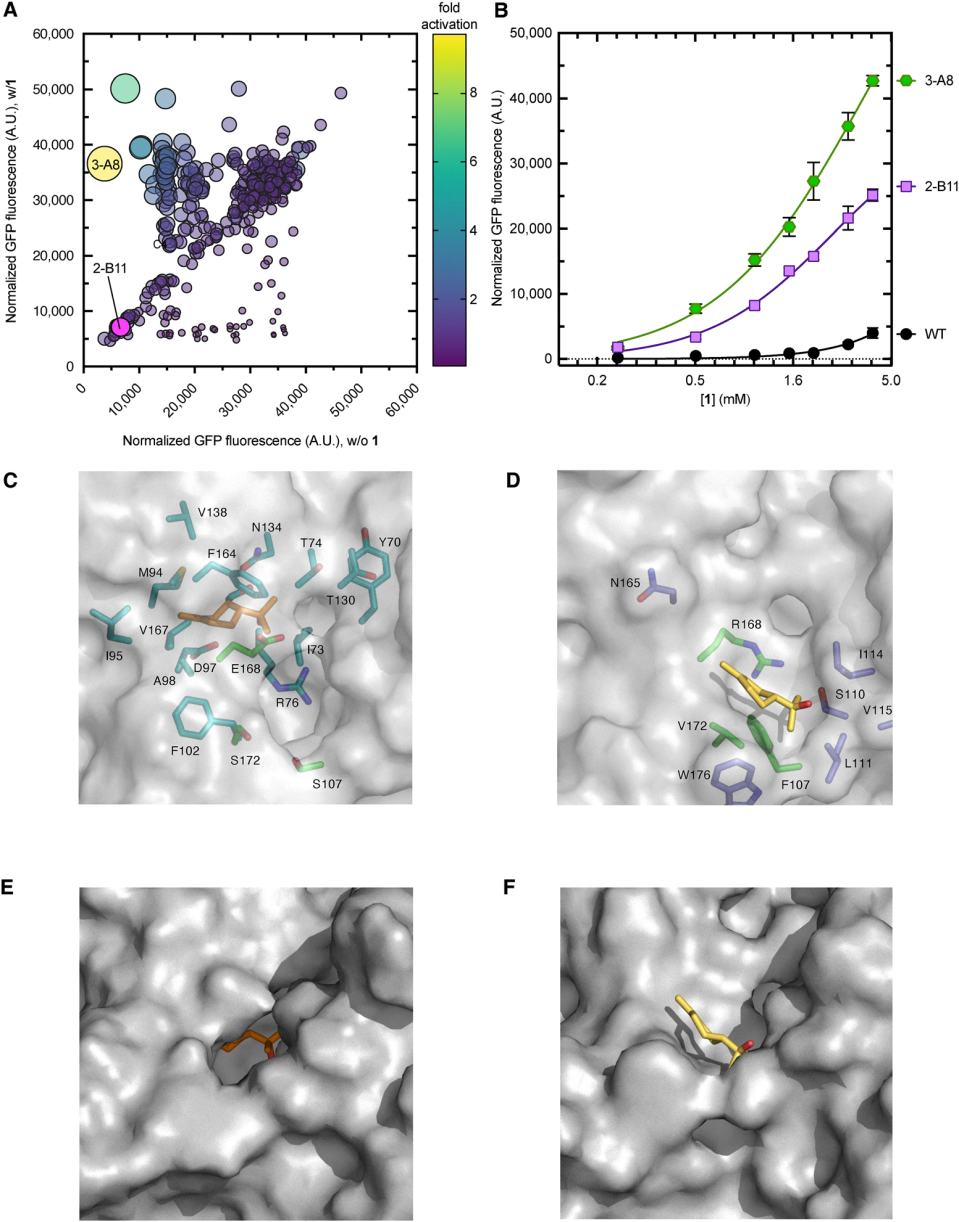
Performance of wild-type and third-generation CymR variants with 1. (A) Responses of individual members of the F107X/R168E/V172S site saturation library with 1 (0.5 mM) and without 1. The relative size of the bubble corresponds to the fold activation of the library member with respect to the parent (2-B11) strain. The relative size of the bubble corresponds to the fold activation of the library variants with respect to the 2-B11 strain. The library member corresponding to the yellow bubble was used for further analysis. (B) Dose–response curves of WT, 2-B11, and 3-A8 with varying concentrations of 1. Error bars (where visible) represent the standard error of the mean (*n* = 3). (C) Model of CymR 3-A8 with 1 docked. Residues targeted for saturation mutagenesis are shown as green sticks. Residues within 5 Å of 1 are shown as cyan sticks. (D) Model of wild-type CymR (same view as D) with 1 docked. Residues targeted for saturation mutagenesis are shown as green sticks. Residues within 5 Å of 1 are shown as blue sticks. (E) Surface of CymR 3-A8 (rotated compared to C) with no transparency to highlight the cavity. (F) Surface of wild-type CymR (same view as E) with no transparency to highlight the shallow 1-binding pocket.

Given that the three-site saturation library was not exhaustively screened but still returned an improved variant with a substitution at Phe107, saturation mutagenesis at Phe107 in the double-site hits (that did not target Phe107) could further improve fold-activation. To test this, the top-performing double mutant, 2-B11 (R168E/V172S), was subjected to saturation mutagenesis at Phe107. Following screening of ∼370 library members with 0.5 mM 1 and subsequent retesting of the initial hits, one library members with improved fold-activation compared to the parent 2-B11 was confirmed: 3-A8 (F̲1̲0̲7̲S̲/R168E/V172S, Fig. 4A). Following analysis of its dose–response curve (Fig. 4B), the dynamic range of 3-A8 was higher than that of any other variant tested, across the entire concentration range of 1 (Fig. 4B and 3B). This resulted in the highest dynamic range (43 000 ± 800) and fold activation (3.5 ± 0.05) observed so far (Fig. 3C). Overall, 3-A8 is the best 1-biosensor strain characterized in this study, clearly demonstrating the impact of introducing F107S to 2-B11. Each of the contributing single amino acid mutations shows very poor activation by 1 and is indistinguishable from the wild-type CymR (Fig. S2C, SI). Combined with the dose response analysis of 2-B11, these results demonstrate that all three mutations contribute to 3-A8's performance. The replacement of bulky hydrophobic residues with smaller, polar ones in the putative ligand-binding pocket of 3-A8 likely reshapes the pocket to better stabilize 1 binding (Fig. 4C). Indeed, after docking 1 with a 3-A8 AlphaFold model, the mutations in 3-A8 appear to open a cavity, enabling 1 to penetrate deeper into the CymR ligand binding pocket than possible in the wild-type CymR (Fig. 4C–F), in a position closer to the putative cumate binding site (Fig. 2C). This supports the original hypothesis that such substitutions would enhance effector accommodation and interactions with CymR. These results confirm the effectiveness of stepwise engineering for improving CymR sensitivity to 1. Since an artificial 1-biosynthetic pathway (Fig. 1A) can likely produce concentrations relevant to the operational range of the 3-A8 biosensor strain, this system is well-suited for guiding the development of enhanced production strains.

### Profiling the specificity and performance of engineered CymR variants with other monoterpenes

**Fig. 5:**
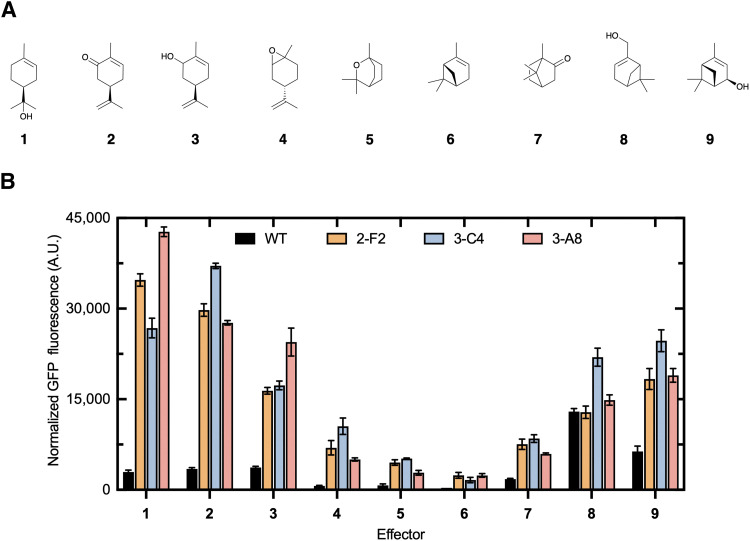
Effector profile of the wild-type and engineered CymR biosensor strains. (A) Compounds used for the characterization of biosensor constructs: 1, (*R*)-(-)-carvone (2), (-)-carveol (3), and (-)-limonene oxide (4). Bicyclic monoterpenes: eucalyptol (5), (-)-α-pinene (6), camphor (7), myrtenol (8), and (*S*)-*cis*-verbenol (9). (B) Relative sfGFP fluorescence (corrected by subtracting fluorescence with no effector) of wild-type, 2-F2, 3-C4, and 3-A8 with nine different monoterpenes at 4 mM. Error bars represent the standard error of the mean (*n* = 3).

A panel of eight additional terpenes (2–9, Fig. 5A) was selected to identify the effector structural features required for efficient de-repression and to determine whether the engineered CymR biosensor could be useful for detecting other industrial or clinically relevant terpenes. The effector promiscuity of the wild-type CymR and variants 2-F2, 3-C4, and 3-A8 across nine monoterpenes (1–9, Fig. 5A) was evaluated by measuring the fluorescence output at a fixed terpene concentration. The wild-type biosensor showed the lowest overall fluorescence, consistent with its limited responsiveness to oxygenated monoterpenes (Fig. 5B). In contrast, the engineered variants exhibited marked improvements in activation, demonstrating that stepwise engineering successfully expanded the effector scope beyond the native ligand profile. Among all tested variants, 3-A8 emerged as the top performer, displaying the strongest and most selective response to 1, with a 20-fold increase in fluorescence relative to the wild type. Variant 3-C4 also showed exceptional activity, achieving a 16-fold increase with 1 and strong activation by 3–5, reflecting a broader yet potent effector recognition profile. The 2-F2 variant showed moderate improvement, producing smaller but consistent gains across the terpene panel. Together, these results highlight 3-A8 and 3-C4 as the most responsive and adaptable biosensor variants within the engineered library (Fig. 5B). Notably, the bicyclic monoterpene 6 elicited the lowest response among all variants, likely due to the absence of a hydroxyl group and its lower polarity compared to the other tested compounds in this panel. This observation supports the notion that the biosensor preferentially recognizes hydroxylated, monocyclic monoterpenes, consistent with its engineered function toward 1. Overall, these data indicate that the mutations primarily enhance dynamic range while preserving a preference for 1, with 3-C4 emerging as the most promiscuous variant.

### Accessing 1*via* the artificial ADH pathway in *E. coli*

**Fig. 6:**
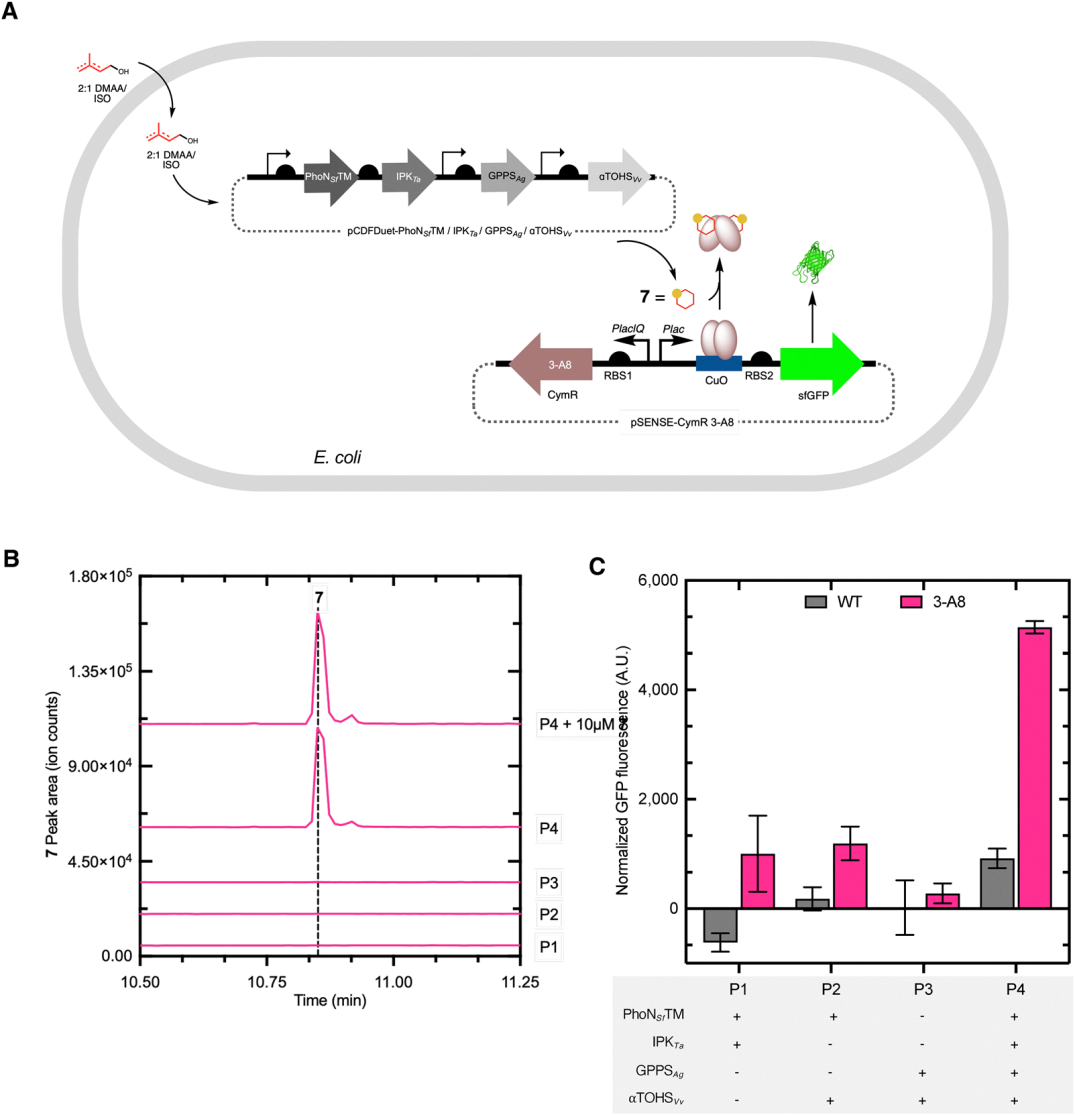
Biosynthesis and detection of 1 in an engineered *E. coli* strain. (A) Scheme showing the four-enzyme pCDFDuet vector consisting of four modules: the ADH module (PhoN_Sf_ and IPK_Ta_); the linear precursor module (GPPS_Ag_); and the cyclization module (αTOHS_Vv_). Production of 1 is monitored by the engineered CymR 3-A8 biosensor (bottom plasmid). (B) Extracted ion count (EIC) chromatograms of 1 (*m*/*z* = 136) determined by GC-MS analysis of the extracted culture media. Error bars represent the standard error of the mean (*n* = 3). (C) The normalized fluorescence output of the full pathway (P4) and deletion strains (P1–P3) using the wild-type and the engineered CymR 3-A8 biosensors.

In this study, we sought to utilize our ADH pathway^27^ in combination with two additional enzymes to produce 1 (Fig. 6A). As a proof-of-concept of the applicability of an *in vivo* biosensor, we also aimed to integrate the evolved 3-A8 biosensor with the minimized pathway to demonstrate *in situ* detection of compound 1. Following protein expression of the three modules (Fig. S4, SI), a 2 : 1 mixture of DMAA/ISO was fed to the *E. coli* strain, and 1 formation was monitored using gas chromatography-mass spectrometry (GC-MS) analysis of the culture extracts. The production of 1 was observed at 136 *m*/*z*, indicating the expected loss of H_2_O (18 *m*/*z*) from the molecular ion (154 *m*/*z*) (Fig. 6B),^63^ and the mass ions consistent with 1 co-eluted with an authentic commercial standard (Fig. 6B). Additionally, the fragmentation of the strain produced 1 was consistent with the standard (Fig. S5A, SI). Notably, 1-production was not observed in a series of control strains (P1–P3) that lacked key genes, including deletion of GPPS/αTOHS (P1), IPK/GPPS (P2), and PhoN/IPK (P3) (Fig. 6B). Using a GC-MS standard curve of 1, the concentration recovered from the culture was ∼150 µM (23.1 mg L^−1^) (Fig. S5B and C, SI). For comparison, using the 3-A8-based dose–response curve (Fig. 4B), the titer of 1 inside the cells was estimated to be ∼300 µM (42.6 mg L^−1^). Given that the biosensor strain differs significantly from the production strain, the difference in estimated titer between the two approaches is not unexpected. Although the estimated titer of 1 represents ∼6% of the theoretical yield from DMAA/ISO, the achieved concentrations exceed the observed titers in microbial hosts and approach those reported for optimized *S. cerevisiae* strains under fed-batch conditions (∼22 mg L^−1^).^18^ These findings highlight the efficiency of the ADH module in supplying isoprenoid precursors and demonstrate its utility as a compact, high-performance route for monoterpene biosynthesis, before large-scale optimization. Furthermore, the system operates within the lower range of the 3-A8 dose–response curve, suggesting that further improvements in 1 production are readily detectable by the biosensor. Comparable advances in ADH pathway optimization have achieved drimenol titers of 398 mg L^−1^,^29^ indicating a significant biosynthetic capacity. While the engineered strain successfully produced 1, the pathway is likely impacted by multiple contributing rate-limiting steps. Any of the four heterologous enzymes, particularly those responsible for phosphorylation and cyclization, may constrain total flux. Further improvement will therefore likely benefit from systematic mutagenesis of the pathway and would be best facilitated with a genetically encoded 1-biosensor.

### Application of the engineered biosensor for the *in-situ* detection of 1

We set out to utilize the engineered CymR variant 3-A8 to report the biosynthesis of 1 in *E. coli*. A two-plasmid system was employed: the first plasmid harbors the heterologous pathway for 1-biosynthesis, while the second plasmid serves as the detection module, harboring the 3-A8 biosensor circuit (Fig. 6A). Together, these two plasmids enable simultaneous biosynthesis and *in vivo* detection of 1 in *E. coli*. The fluorescence output of the dual plasmid production/detection strain was determined after protein expression and the addition of a 2 : 1 mixture of 5 mM DMAA/ISO to the culture. As anticipated, the control strains that lacked key genes displayed low (<2000 RFU) fluorescence output (Fig. 6C). In the presence of all four genes in the P4 strain (PhoN_SF_TM/IPK_Ta_/GPPS_Ag_/αTOHS_Vv_), a significant fluorescence response (*p* < 0.05) was observed compared to the deletion strains (P1–P3) when the 3-A8 biosensor was used (Fig. 6B), confirming that the CymR 3-A8 biosensor detects 1 formation only when the entire pathway is present (Fig. 6C). This observation is consistent with the GC-MS chromatograms (Fig. 6B), where a clear 1 peak was detected only with the complete pathway, strain P4. The statistically significant fluorescence output of the full pathway strain highlights the ability of the CymR 3-A8 biosensor to report 1-production *in situ* and demonstrates the effectiveness of the minimized biosynthetic pathway. The fluorescence output of the CymR 3-A8 variant in the production strain corresponded to the lower to mid region of the dose response curve (<0.5 mM 1) of the 3-A8 biosensor strain (Fig. 4B), consistent with a moderate level of *in situ*1 accumulation.

The fluorescence outputs of all the deletion strains (P1–P3), using the more sensitive 3-A8 biosensor, are low and statistically indistinguishable from each other, as expected given the lack of dedicated enzymes required for 1-production (Fig. 6B and C). The measurable, albeit statistically indistinguishable, fluorescence output across the deletion strains P1–P3 with 3-A8 suggests that perhaps the biosensor is detecting 1 production at a concentration lower than the GC-MS detection limit (unlikely) or that the biosensor is demonstrating leakiness in the two-plasmid production/detection strain that was not expected based on the one-plasmid biosensor strains (more likely). Furthermore, the 3-A8 biosensor exhibited higher fluorescence output than wild-type CymR across all tested strains (Fig. 6B), confirming its enhanced detection ability.

## Conclusion

This work addresses the lack of biosensors capable of detecting hydroxylated monoterpenes such as 1. We successfully generated a novel transcription factor–based biosensor derived from the wild-type CymR regulator and applied a focused directed evolution approach to improve its performance. Using a small library and a streamlined screening workflow, only three rounds of screening, covering just 1870 colonies, yielded variants with a 22-fold improvement in dynamic range. Among these, CymR variant 3-A8 demonstrated superior sensitivity toward 1. These results confirm that targeted manipulation of the transcription factor ligand-binding pocket can effectively enhance signal response and broaden effector specificity, establishing a versatile tool to enable high-throughput pathway engineering. It is anticipated that related transcription factors could also be improved *via* a similar approach. The integration of this biosensor into an *E. coli* strain harboring a four-enzyme pathway confirmed the production of 1 and its detection. Furthermore, the 3-A8 biosensor platform distinguished the full pathway from various deletion strains. The coupling of the 1-scaffold assembly module with the ADH pathway provides an efficient, modular alternative for the biosynthesis of monocyclic terpene alcohols. This biosynthetic route eliminates the need for labor-intensive chemical synthesis of alkyl pyrophosphate intermediates, providing a flexible platform for isoprenoid-based natural product biosynthesis. The results provide insights into transcription factor structure–function relationships and reinforce the value of compact, modular biosensor/production designs for monitoring and optimizing terpene biosynthesis. Ultimately, the 3-A8 platform offers a broadly applicable model for linking product formation with genetic diversification, enabling rapid screening of high-performing variants and facilitating the rational advancement of synthetic metabolic pathways for complex natural products.

## Author contributions

C. A. O. and G. J. W. conceived the project. C. A. O. conducted all the studies. I. A. contributed to the design, construction, and testing of the production strain and deletion mutants. All authors analyzed the data. The manuscript was written by C. A. O. and G. J. W., with contributions and proofreading from all authors.

## Conflicts of interest

There are no conflicts to declare.

## Supplementary Material

CB-007-D5CB00310E-s001

## Data Availability

The data supporting this article have been included as part of the supplementary information (SI). Supplementary information: Tables S1 and S2, Fig. S1–S5, and further experimental details. See DOI: https://doi.org/10.1039/d5cb00310e. Raw data files are freely available upon request.
