# Supplementary material for: Development of a transcription factor-based biosensor strain for reporting α-terpineol production via the alcohol-dependent hemiterpene pathway in Escherichia coli
Source: RSC Chem Biol. 2026 Feb 18;7(4):695–707. doi: 10.1039/d5cb00310e (PMC12947842; doi:10.1039/d5cb00310e)
Supplement: CB-007-D5CB00310E-s001 [file CB-007-D5CB00310E-s001.pdf]

Supplementary Information (SI) for RSC Chemical Biology.
This journal is © The Royal Society of Chemistry 2026

Development of a transcription factor-based biosensor strain for reporting α-terpineol production via the alcohol-dependent hemiterpene pathway in *Escherichia coli*

Catherine A. Odhiambo[1], Isaac A. Ali[1,2], and Gavin J. Williams[1,3]*

[1] Department of Chemistry, NC State University, Raleigh, North Carolina 27695, United States

[2] Present address: Human Immunology Section, Vaccine Research Center, National Institute of Allergy and Infectious Diseases, National Institutes of Health, Bethesda, Maryland 20814, United States.

[3] Comparative Medicine Institute, NC State University, Raleigh, North Carolina 27695, United States

* Corresponding author, gjwillia@ncsu.edu

Table of Contents

Supplementary Methods **2**

Construction of pSENSE-CymR biosensor circuit plasmid *2*

Site-directed mutagenesis of CymR 2

Saturation mutagenesis of CymR 3

Construction of αTOH production plasmids *4*

Site-directed mutagenesis of PhoN variants 7

Preparation of a dual-plasmid for the in-situ detection of 1 .... *8*

Supplementary Tables **9**

Supplementary Table S1. *Sequences (5'—3') of DNA constructs.* 9

Supplementary Table S2: *Oligonucleotides (5'—3') used in this study. Mutations are in lower case.* 13

Supplementary Figures **15**

Supplementary Figure S1. *The plasmid map of pSENSE3-CymR-sfGFP (top) and pSENSE3-CymR-sfGFP (bottom).* 15

Supplementary Figure S2. *Performance of wild-type and CymR variants with **1**.* 16

Supplementary Figure S3. *3D structures of **1** and p-cumate.* 17

Supplementary Figure S4. *SDS-PAGE analysis of **1**-production and deletion strains. Lane 1, protein marker; Lane 2, whole cell lysate of E. coli BL21(DE3) pCDFDuet-PhoN$_{Sf}^{TM}$-IPK$_{Ta}$; Lane 3, E. coli BL21(DE3) pCDFDuet-PhoN$_{Sf}^{TM}$-GPPS$_{Ag}$; Lane 4, E. coli BL21(DE3) pCDFDuet-PhoN$_{Sf}^{TM}$-GPPS$_{Ag}$-αTOHS$_{Vv}$; and Lane 5, E. coli BL21(DE3) pCDFDuet-PhoN$_{Sf}^{TM}$-IPK$_{Ta}$-GPPS$_{Ag}$-αTOHS$_{Vv}$. The expected MW based on the protein sequence is shown in brackets.* 18

Supplementary Figure S5. *GC–MS identification and quantification of **1** from culture extracts.* 19

Supplementary References **20**

Supplementary Methods

Construction of pSENSE-CymR biosensor circuit plasmid

The plasmid pSENSE-CymR biosensor was constructed by cloning the operator sequence cuO into pSENSE2 [1]using primers 1-2 (**Supplementary Table S2**) and the *Avr*II and *Spe*I restriction sites to create pSENSE3. The CymR fragment, which was codon-optimized for expression in *E. coli*, was amplified using primers 3-4 (**Supplementary Table S2**). The amplified products were analyzed on a 0.8 % agarose gel, extracted using a Monarch Gel Extraction Kit (New England Biolabs), digested with *Avr*II and *Spe*I, and ligated for 18 h at 16 °C. The mixture was subsequently transformed into chemically competent *E. coli* DH5α cells. The transformation mixture was plated on LB agar containing ampicillin (100 µg/mL) and incubated for 16 h at 37 °C. A single colony was cultured in 3 mL of ampicillin-treated LB for 16 h at 37 °C with shaking at 250 rpm, followed by plasmid isolation using the Monarch Spin Plasmid Miniprep Kit according to the instructions.

Site-directed mutagenesis of CymR

Site-directed mutagenesis for the construction of CymR variants was performed by PCR in a total volume of 25 µL, containing Q5 Hot Start High Fidelity 2X master mix (12.5 µL), DNase-free water (9 µL), and a forward and reverse primer mixture (2.5 µL, 10 µM). Single point mutations were introduced separately in the *CymR* gene, using 5 ng DNA template and primers 5-10 (**Supplementary Table S2**). The annealing temperatures were as follows: 98 °C, 30 s; [98 °C, 10 s; [56 °C (F107A), 64 °C (R168L), 68 °C (V172A), 15 s]; 72 °C, 2.4 min] cycle step 2-4 25X, 72°C, 10 min, and hold at 4 °C. The PCR product was digested with *Dpn*I at 37 °C for 1 h, then at 80 °C for 20 min, analyzed on a 0.8 % agarose gel, and extracted using a Monarch Gel Extraction Kit. The ligation procedure was carried out in a total volume of 10 µL containing T4 ligase (1 µL), T4 ligase buffer (1 µL), and T4 polynucleotide kinase (PNK) (0.5 µL) for 1 h at 25 °C, and the mixture was transformed into chemically competent *E. coli* DH5α cells. The transformation mixture was plated on LB agar containing ampicillin (100 µg/mL) and incubated for 16 h at 37 °C. A single colony was cultured in 3 mL of ampicillin-treated LB for 16 h at 37 °C with shaking at 250 rpm, followed by plasmid isolation using the Monarch Spin Plasmid Miniprep Kit according to the instructions. DNA sequences of the plasmids from individual colonies confirmed that the correct mutation was obtained.

The pSENSE3-CymR-F107A (5 ng) plasmid served as a template for introducing a second point mutation at Arg168 and Val172 using primers 7-8 and 9-10 (**Supplementary Table S2**), respectively, resulting in pSENSE3-CymR-F107A/R168L and pSENSE3-CymR-F107A/V172A, respectively. The plasmid pSENSE3-CymR-R168L/V172A was created using primers 11-12 (**Supplementary Table S2**) using pSENSE3-CymR wild-type template (5 ng). To generate the CymR variant pSENSE3-CymR-F107A/R168L/V172A, site-directed mutagenesis was performed, utilizing primers 5-6 (**Supplementary Table S2**) to introduce a mutation at Phe107 using pSENSE3-CymR-R168L/V172A as template. Likewise, a blunt-end PCR procedure to create CymR double and triple mutants was performed using the PCR parameters and reagents established earlier. The annealing temperature for R168L/V172A was set at 66 °C. The PCR product was digested with *Dpn*I at 37 °C for 1h, then at 80 °C for 20 min, analyzed on a 0.8 % agarose gel, and extracted using a Monarch Gel Extraction Kit. The ligation was

carried out in a total volume of 10 µL containing T4 ligase (1 µL), T4 ligase buffer (1 µL), and T4 PNK (0.5 µL) for 1 h at 25 °C, and the reaction was transformed into chemically competent *E. coli* DH5α cells. The transformation mixture was plated on LB agar containing ampicillin (100 µg mL^{-1}) and incubated for 16 h at 37 °C. A colony was cultured in 3 mL of ampicillin-treated LB for 16 h at 37 °C with shaking at 250 rpm, followed by plasmid isolation using the Monarch Spin Plasmid Miniprep Kit according to the instructions. DNA sequences of the plasmids from individual colonies confirmed that the correct mutation was obtained.

Saturation mutagenesis of CymR

Second generation, 2-site saturation mutagenesis

The pSENSE3-CymR wild-type (10 ng) was subjected to saturation mutagenesis at the positions Arg168 and Val172 with partially degenerate NNK codon primers 13-14 (**Supplementary Table S2**). The PCR procedure was conducted to a total volume of 25 µL containing Q5 Hot Start High Fidelity 2X master mix (12.5 µL), DNase-free water mix (9 µL), and forward and reverse primer mixture (2.5 µL, 10 µM) (**Supplementary Table S2**). The PCR parameters temperatures were as follows: 98 °C, 30 s; [98 °C, 10 s; 66 °C, 15 s; 72 °C, 2.4 min] cycle step 2-4 25X, 72 °C, 10 min, and hold at 4 °C. The PCR product was digested with *Dpn*I at 37 °C for 1h, then at 80 °C for 20 min, analyzed on a 0.8% agarose gel, and extracted using a Monarch Gel Extraction Kit. The ligation procedure was performed in a total volume of 10 µL containing T4 ligase (1 µL), T4 ligase buffer (1 µL), and T4 PNK (0.5 µL) for 1 h at 25°C, and the mixture was transformed into chemically competent *E. coli* TOP10 cells. The remaining 25 µL was cultured in 4 mL of ampicillin-treated LB for 16 h at 37 °C, with shaking at 250 rpm. The 500 µL of 50% glycerol (v/v) and 500 µL of culture were stored at -80 °C. The DNA plasmid was isolated from the leftover culture and stored for later use. DNA sequences of the plasmids from ten individual colonies were sequenced to confirm substitutions at the desired codon.

Second generation, 3-site saturation mutagenesis

The pSENSE3-CymR wild-type (10 ng) was subjected to saturation mutagenesis at the positions Arg168 and Val172 with partially degenerate NNK codon primers 13-14 (**Supplementary Table S2**). The PCR procedure was conducted to a total volume of 25 µL containing Q5 Hot Start High Fidelity 2X master mix (12.5 µL), DNase-free water mix (9 µL), and forward and reverse primer mixture (2.5 µL, 10 µM) (**Supplementary Table S2**). The PCR parameters temperatures were as follows: 98 °C, 30 s; [98 °C, 10 s; 66 °C, 15 s; 72 °C, 2.4 min] cycle step 2-4 25X, 72 °C, 10 min, and hold at 4 °C. The PCR product was digested with *Dpn*I at 37 °C for 1h, then at 80 °C for 20 min, analyzed on a 0.8 % agarose gel, and extracted using a Monarch Gel Extraction Kit. The eluted (3 µL, 70 ng/µL) PCR product was subject to an additional round of saturation mutagenesis at position Phe107 with partially degenerate NNK codon primers 15-16 (**Supplementary Table S2**). An annealing temperature of 71 °C was used for saturation mutagenesis at Phe107. The PCR product was digested with *Dpn*I at 37 °C for 1h, then at 80 °C for 20 min, analyzed on a 0.8% agarose gel, and extracted using a Monarch Gel Extraction Kit. The ligation was performed to a total volume of 10 µL containing T4 ligase (1 µL), T4 ligase buffer (1 µL), and T4 PNK (0.5 µL) for 1 h at 25 °C, and the reaction was transformed into chemically competent *E. coli* TOP10 cells. A 25 µL volume of the remaining transformation mixture was plated on LB agar containing ampicillin (100 µg/mL) and

incubated for 16 h at 37 °C. The remaining 25 µL was cultured in 4 mL of ampicillin-treated LB for 16 h at 37 °C, with shaking at 250 rpm. The 500 µL of 50% glycerol (v/v) and 500 µL of culture were stored at -80 °C. The plasmid was isolated from the leftover culture and stored for later use. DNA sequences of the plasmids from ten individual colonies were sequenced to confirm the substitutions at the desired codon.

Third-generation, saturation mutagenesis

pSENSE3-CymR-R168E/V172S (10 ng) was subjected to saturation mutagenesis at position Phe107 with partially degenerate NNK codon primers 15-16 (**Supplementary Table S2**). The PCR procedure was conducted to a total volume of 25 µL containing Q5 Hot Start High Fidelity 2X master mix (12.5 µL), DNase-free water mix (9 µL), and forward and reverse primer mixture (2.5 µL, 10 µM (**Supplementary Table S2**). The PCR parameters temperatures were as follows: 98 °C, 30 s; [98 °C, 10 s; 71 °C, 15 s; 72 °C, 2.4 min] cycle step 2-4 25X, 72 °C, 10 min, and hold at 4 °C. The PCR product was digested with *Dpn*I at 37 °C for 1h, heated to 80 °C for 20 min, analyzed on a 0.8% agarose gel, and extracted using a Monarch Gel Extraction Kit. The ligation was performed in a total volume of 10 µL, containing T4 ligase (1 µL), T4 ligase buffer (1 µL), and T4 PNK (0.5 µL), and incubated for 1 h at 25 °C. The reaction was then transformed into chemically competent *E. coli* TOP10 cells. The remaining 25 µL was added to 4 mL of ampicillin-treated LB and incubated for 16 h at 37 °C with shaking at 250 rpm. The 500 µL of 50% glycerol (v/v) and 500 µL of culture were stored at -80 °C. The DNA plasmid was isolated from the leftover culture and stored for later use. DNA sequences of the plasmids from ten individual colonies were sequenced to confirm the substitutions at the desired codon .

Construction of αTOH production plasmids

Construction of strain P1 (ΔGPPS/αTOHS): pCDFDuet-PhoN/IPK

A pCDFDuet-1 plasmid was linearized by PCR amplification using primers 17-18 (**Supplementary Table S4**) to obtain a backbone for the assembly of PhoN/IPK (ADH). The ADH insert fragment was amplified from pETDuet-ADH [2]using primers 19-20 (**Supplementary Table S4).** In a total volume of 25 µL containing 5X GC Phusion DNA polymerase buffer (10 µL), DNase-free water (10 µL), forward/reverse primer mixture (2.0 µL, 10 µM) (**Supplementary Table S4**), template DNA (1 µL, 50 ng/µL), dNTPs (0.5 µL, 2.5 mM each dNTP), and Phusion High Fidelity DNA Polymerase (0.5 µL). The PCR parameters temperatures were as follows: 98 °C, 30 s; [98 °C, 15 s; 60 °C, 15 s; 72 °C, 1.0 min (ADH) and 2.0 min (pCDFDuet)] cycle step 2-4 34X, 72 °C, 10 min, and hold at 4 °C. The PCR product was digested with *Dpn*I at 37 °C for 1 h, then heated to 80 °C for 20 min, and analyzed on a 0.8% agarose gel. The gel was extracted using a Monarch Gel Extraction Kit.

A 1:4 vector and PhoN/IPK insert were assembled, using 10 µL of HiFi DNA Assembly Master Mix and water to adjust the volume. The assembly was performed at 50 °C for 1 h. An aliquot (4 µL) of the Gibson Assembly was transformed into chemically competent *E. coli* DH5α cells and recovered for 1 h. The transformation mixture was plated on LB agar containing streptomycin (50 µg/mL) and incubated for 16 h at 37 °C. Four colonies were cultured in four separate 3 mL tubes of streptomycin-treated LB medium for 16 hours at 37 °C, with shaking at 250 rpm. This was followed by plasmid

isolation using the Monarch Spin Plasmid Miniprep Kit according to the manufacturer's instructions. DNA sequences of the plasmids from four individual colonies were sequenced to confirm assembly for pCDFDuet-PhoN/IPK.

Construction of P2 (ΔIPK/GPPS): pCDFDuet-PhoN/αTOHS

The plasmid pCDFDuet-IPK/αTOHS was constructed by cloning the RBS-PhoN fragment from pET28a-PhoN into MC1 of pCDFDuet-GPPs/αTOHs via Gibson Assembly. Primers 29-32 were used for the insert fragment and linear vector amplifications (**Supplementary Table S4**). The PCR procedure was conducted in a total volume of 25 µL, containing Q5 Hot Start High Fidelity 2X master mix (12.5 µL), DNase-free water (9 µL), and a forward and reverse primer mixture (2.5 µL, 10 µM). The PCR parameters temperatures were as follows: 98 °C, 30 s; [98 °C, 10 s; 62 °C, 15 s; 72 °C, 2.7 min (backbone) and 40 s (insert)] cycle step 2-4 25X, 72 °C, 10 min, and hold at 4 °C. The PCR product was digested with *Dpn*I at 37 °C for 1h, then at 80 °C for 20 min, analyzed on a 0.8% agarose gel, and extracted using a Monarch Gel Extraction Kit. A 1:4 ratio of backbone and inset fragments was assembled by leveraging the NEBuilder HiFi DNA Assembly Kit. An aliquot (4 µL) of the Gibson Assembly was transformed into chemically competent *E. coli* DH5α cells and recovered for 2 h. The transformation mixture was plated on LB agar containing streptomycin (50 µg/mL) and incubated for 16 h at 37 °C. Four colonies were cultured in four separate 3 mL tubes of streptomycin-treated LB medium for 16 h at 37 °C, with shaking at 250 rpm. This was followed by plasmid isolation using the Monarch Spin Plasmid Miniprep Kit according to the manufacturer's instructions. DNA sequences from the plasmids of four individual colonies were sequenced to confirm the assembly.

Construction of strain P3 (ΔPhoN/IPK): pCDFDuet-GPPS-αTOHS

The GPPs and αTOHs, which were codon-optimized for expression in *E. coli,* were amplified using primers 21-24 (**Supplementary Table S4**) into the multicloning sites (MC1 and MC2) of pCDFDuet. The PCR procedure was conducted in a total volume of 25 µL, containing Q5 Hot Start High Fidelity 2X master mix (12.5 µL), DNase-free water (9 µL), and a forward and reverse primer mixture (2.5 µL, 10 µM). The PCR parameters temperatures were as follows: 98 °C, 30 s; [98 °C, 10 s; 61 °C (GPPs), 65 °C (αTOHs), 15 s; 72 °C, 47 s (GPPs) and 1.0 min (αTOHs)] cycle step 2-4 25X, 72 °C, 10 min, and hold at 4 °C. The PCR product was digested with *Dpn*I at 37 °C for 1 h, then heated to 80 °C for 20 min, and analyzed on a 0.8% agarose gel. The gel was extracted using a Monarch Gel Extraction Kit.

The pCDFDuet-1 plasmid backbone was linearized by PCR using vector-specific primer pairs 25-26 (**Supplementary Table S4**) for assembly of GPPS. A total volume of 25 µL containing Q5 Hot Start High Fidelity 2X master mix (12.5 µL), DNase-free water mix (9 µL), and forward and reverse primer mixture (2.5 µL, 10 µM). The PCR parameters temperatures were as follows: 98 °C, 30 s; [98 °C, 10 s; 59 °C, 15 s; 72 °C, 2 min] cycle step 2-4 25X, 72 °C, 10 min, and hold at 4 °C. The PCR product was digested with *Dpn*I at 37 °C for 1 h, followed by a 20-minute incubation at 80 °C, and then analyzed on a 0.8% agarose gel. The gel was extracted using a Monarch Gel Extraction Kit. A 1:4 vector and GPPs insert fragment were assembled by leveraging the NEBuilder HiFi DNA Assembly Kit. An aliquot (4 µL) of the Gibson Assembly was

transformed into chemically competent *E. coli* DH5α cells and recovered for 1 h. The transformation mixture was plated on LB agar containing streptomycin (50 µg/mL) and incubated for 16 h at 37 °C. Four colonies were cultured in four separate 3 mL tubes of streptomycin-treated LB medium for 16 hours at 37 °C, with shaking at 250 rpm. This was followed by plasmid isolation using the Monarch Spin Plasmid Miniprep Kit according to the manufacturer's instructions. DNA sequences from the plasmids of four individual colonies were sequenced to confirm the assembly.

A pCDFDuet-GPPS plasmid was linearized with vector-specific primer pairs 27-28 (**Supplementary Table S4**) to obtain a vector for the assembly of αTOHS. A total volume of 25 µL containing Q5 Hot Start High Fidelity 2X master mix (12.5 µL), DNase-free water mix (9 µL), and forward and reverse primer mixture (2.5 µL, 10 µM). The PCR parameters temperatures were as follows: 98 °C, 30 s; [98 °C, 10 s; 65 °C, 15 s; 72 °C, 2.3 min] cycle step 2-4 25X, 72 °C, 10 min, and hold at 4 °C. The PCR product was digested with *Dpn*I at 37 °C for 1 h, followed by an incubation at 80 °C, and then analyzed on a 0.8% agarose gel. The gel was extracted using a Monarch Gel Extraction Kit. A 1:4 ratio of vector and αTOHS insert was assembled by leveraging the NEBuilder HiFi DNA Assembly Kit. An aliquot (4 µL) of the Gibson Assembly was transformed into chemically competent *E. coli* DH5α cells and recovered for 2 h. The transformation mixture was plated on LB agar containing streptomycin (50 µg/mL) and incubated for 16 h at 37 °C. Four colonies were cultured in four separate 3 mL tubes of streptomycin-treated LB medium for 16 h at 37 °C, with shaking at 250 rpm. This was followed by plasmid isolation using the Monarch Spin Plasmid Miniprep Kit according to the manufacturer's instructions. DNA sequences from the plasmids of four individual colonies were sequenced to confirm the assembly. A 1:4 vector and insert were assembled by leveraging the NEBuilder HiFi DNA Assembly Kit.

Construction of strain P4 (full pathway): pCDFDuet-PhoN/IPK(ADH)/GPPS/αTOHS

First, pCDFDuet-PhoN/GPPS/αTOHS was constructed by cloning the RBS-PhoN insert fragment to GPPS of pCDFDuet-GPPS/αTOHS using primers 33-36 (**Supplementary Table S4**). The PCR was conducted in a total volume of 25 µL, containing Q5 Hot Start High Fidelity 2X master mix (12.5 µL), DNase-free water (9 µL), and a forward and reverse primer mixture (2.5 µL, 10 µM). The PCR parameters temperatures were as follows: 98 °C, 30 s; [98 °C, 10 s; 62 °C, 15 s; 72 °C, 3.0 min (backbone) and 42 s (insert)] cycle step 2-4 25X, 72 °C, 10 min, and hold at 4 °C. The PCR product was digested with *Dpn*I at 37 °C for 1h, 80 °C for 20 min, analyzed on a 0.8% agarose gel, and extracted using a Monarch Gel Extraction Kit. A 1:4 ratio of backbone and insert fragments was assembled by leveraging the NEBuilder HiFi DNA Assembly Kit. An aliquot (4 µL) of the Gibson Assembly was transformed into chemically competent *E. coli* DH5α cells and recovered for 2 h. Then, 25 µL of the resuspended transformation mixture was plated on LB agar containing streptomycin (50 µg/mL) and incubated for 16 h at 37 °C. Four colonies were cultured in four separate 3 mL of streptomycin-treated LB for 16 h at 37 °C, with shaking at 250 rpm, followed by plasmid isolation according to the instructions for the Monarch Spin Plasmid Miniprep Kit. DNA sequences from the plasmids of four individual colonies were sequenced to confirm the assembly.

Then, IPK was cloned into the area adjacent to the GPPS of the pCDFDuet-PhoN/GPPS/αTOHS plasmid using primers 37-40 (**Supplementary Table S4**). The PCR reactions were conducted in a total volume of 25 µL, containing Q5 Hot Start

High Fidelity 2X master mix (12.5 µL), DNase-free water (9 µL), and a forward and reverse primer mixture (2.5 µL, 10 µM). The PCR parameters temperatures were as follows: 98 °C, 30 s; [98 °C, 10 s; 66 °C and 62 °C (insert) °C, 15 s; 72 °C, 3.5 min (backbone) and 50 s (insert)] cycle step 2-4 25X, 72 °C, 10 min, and hold at 4 °C. The PCR product was digested with *Dpn*I at 37 °C for 1h, then heated to 80 °C for 20 min, analyzed on a 0.8% agarose gel, and extracted using a Monarch Gel Extraction Kit. A 1:4 ratio of backbone and insert fragments was assembled by leveraging the NEBuilder HiFi DNA Assembly Kit. A 4 µL aliquot of the Gibson Assembly was transformed into chemically competent *E. coli* DH5α cells and recovered for 2 h. 25 µL of the resuspended transformation mixture was plated on LB agar containing streptomycin (50 µg/mL) and incubated for 16 h at 37 °C. Four colonies were cultured in four separate 3 mL of streptomycin-treated LB for 16 h at 37 °C, with shaking at 250 rpm, followed by plasmid isolation according to the instructions for the Monarch Spin Plasmid Miniprep Kit. DNA sequences of the plasmids from four individual colonies were sequenced to confirm pCDFDuet-ADH/GPPS/αTOHS assembly.

Site-directed mutagenesis of PhoN variants

Construction of PhoN$_{E122R/T157K/R160K}$ variants

Plasmids pCDFDuet-PhoN/IPK and pCDFDuet-PhoN/αTOHS were used for site-directed mutagenesis at PhoN positions Thr157 and Arg160, utilizing primers 41-42 (**Supplementary Table S4**). The PCR procedure was conducted in a total volume of 25 µL, containing Q5 Hot Start High Fidelity 2X master mix (12.5 µL), DNase-free water (9 µL), and a forward and reverse primer mixture (2.5 µL, 10 µM). The PCR parameters temperatures were as follows: 98 °C, 30 s; [98 °C, 10 s; 65 °C, 15 s; 72 °C, 3.0 min] cycle step 2-4 25X, 72 °C, 10 min, and hold at 4 °C.

The PCR product was digested with *Dpn*I at 37 °C for 1h, then heated to 80 °C for 20 min, analyzed on a 0.8% agarose gel, and extracted using a Monarch Gel Extraction Kit. The ligation was conducted to a total volume of 10 µL containing T4 ligase (1 µL), T4 ligase buffer (1 µL), and T4 PNK (0.5 µL) for 1 h at 25 °C. A 4 µL aliquot of the Gibson Assembly was transformed into chemically competent *E. coli* DH5α cells and recovered for 2 h. 25 µL of the resuspended transformation mixture was plated on LB agar containing streptomycin (50 µg/mL) and incubated for 16 h at 37 °C. Four colonies were cultured in four separate 3 mL tubes of streptomycin-treated LB medium for 16 h at 37 °C, with shaking at 250 rpm. This was followed by plasmid isolation using the Monarch Spin Plasmid Miniprep Kit according to the manufacturer's instructions. DNA sequences of the plasmids from individual colonies confirmed that the pCDFDuet-PhoN-$_{T157K/R160K}$/IPK and pCDFDuet-PhoN-$_{T157K/R160K}$/αTOHS mutants were obtained.

For the second PCR, pCDFDuet-PhoN-$_{T157K/R160K}$/IPK and pCDFDuet-PhoN-$_{T157K//R160K}$/αTOHS were used for site-directed mutagenesis at Glu122, utilizing primers 43-44 (**Supplementary Table S4**). The PCR procedure was conducted in a total volume of 25 µL, containing Q5 Hot Start High Fidelity 2X master mix (12.5 µL), DNase-free water (9 µL), and a forward and reverse primer mixture (2.5 µL, 10 µM). The PCR parameters temperatures were as follows: 98 °C, 30 s; [98 °C, 10 s; 63 °C, 15 s; 72 °C, 3.0 min] cycle step 2-4 25X, 72 °C, 10 min, and hold at 4 °C.

The PCR product was digested with *Dpn*I at 37 °C for 1 h, then heated to 80 °C for 20 min, and analyzed on a 0.8% agarose gel. The gel was extracted using a Monarch Gel Extraction Kit. The ligation was conducted to a total volume of

10 µL containing T4 ligase (1 µL), T4 ligase buffer (1 µL), and T4 PNK (0.5 µL) for 1 h at 25 °C. A 4 µL aliquot of the Gibson Assembly was transformed into chemically competent *E. coli* DH5α cells and recovered for 2 h. 25 µL of the resuspended transformation mixture was plated on LB agar containing streptomycin (50 µg/mL) and incubated for 16 h at 37 °C. Four colonies were cultured in four separate 3 mL tubes of streptomycin-treated LB medium for 16 h at 37 °C, with shaking at 250 rpm. This was followed by plasmid isolation using the Monarch Spin Plasmid Miniprep Kit according to the manufacturer's instructions. DNA sequences of the plasmids from individual colonies confirmed that the pCDFDuet-PhoN-$_{E122R/T157K/R160K}$/IPK and pCDFDuet-PhoN-$_{E122R/T157K/R160K}$/αTOHS mutants were obtained.

A similar PCR procedure, with a 4 min extension time, was used to introduce three-point mutations at Glu122, Thr157, and Arg160 of the PhoN gene into the pCDFDuet-PhoN/IPK/GPPS/αTOHS.

Preparation of a dual-plasmid for the in-situ detection of 1

The DNA plasmid of the pSENSE3-CymR-3A8 biosensor was transformed into separate 50 µL of *E. coli* BL21(DE3) competent cells that contained the full pathway for **1** production or various deletions (P1-P3) housed in the pCDFDuet vector. The transformation process involved a heat shock step at 42 °C for 50 s, followed by a recovery phase in 950 µL of SOC medium at 37 °C for 2 h while shaking at 250 rpm. After recovery, a 25 µL aliquot of the transformation mixture was plated on LB agar supplemented with a 1:1 ratio of streptomycin and ampicillin at 50 µg/mL. The plates were then incubated for 16 h at 37 °C.

Supplementary Tables

Supplementary Table S1. Sequences (5'—3') of DNA constructs.

**Construct Name**	**DNA Sequence**
pSENSE-CymR3-*sf*GFP<br><br>CymR (**purple**)<br>NCBI: U24215.1<br>CuO (**red**)<br>*sf*GFP (**green**)<br>NCBI: MK301203.1	TTGAGATCCTTTTTTTCTGCGCGTAATCTGCTGCTTGCAAACAAAAAAACCACCGCTACCAGCGGTGGTTTGTTTGCCGGATCAAGAGCTA<br>CCAACTCTTTTTCCGAAGGTAACTGGCTTCAGCAGAGCGCAGATACCAAATACTGTCCTTCTAGTGTAGCCGTAGTTAGGCCACCACTTC<br>AAGAACTCTGTAGCACCGCCTACATACCTCGCTCTGCTAATCCTGTTACCAGTGGCTGCTGCCAGTGGCGATAAGTCGTGTCTTACCGGG<br>TTGGACTCAAGACGATAGTTACCGGATAAGGCGCAGCGGTCGGGCTGAACGGGGGGTTCGTGCACACAGCCCAGCTTGGAGCGAACGA<br>CCTACACCGAACTGAGATACCTACAGCGTGAGCTATGAGAAAGCGCCACGCTTCCCGAAGGGAGAAAGGCGGACAGGTATCCGGTAAG<br>CGGCAGGGTCGGAACAGGAGAGCGCACGAGGGAGCTTCCAGGGGGAAACGCCTGGTATCTTTATAGTCCTGTCGGGTTTCGCCACCTC<br>TGACTTGAGCGTCGATTTTTGTGATGCTCGTCAGGGGGGCGGAGCCTATGGAAAAACGCCAGCAACGCGGCCTTTTTACGGTTCCTGGC<br>CTTTTGCTGGCCTTTTGCTCACATGTTCTTTCCTGCGTTATCCCCTGATTCTGTGGATAACCGTATTACCTCTAGTGTACAGTGATCAAGAC<br>TTCGATACCACCGACCGTACCGGTACTAATCGACGACGGTCGTGTTCGTCGCCTGCCGCAGGGACTCTGCACACCTTAAGCTATTTGTA<br>CAACTCATCCATCCCTCCAGTTGAGTGGCGTCCTTCGGCCCGCTCATACTGTTCTACAATTGTATAGTCTTCGTTATGGGACGTGATGTCC<br>AACTTAATATTAACATTATAGGCACCGGGAAGCTGCACGGGTTTTTTAGCCTTGTAAGTAGTTTTGACCTCAGCATCGTAGTGTCCACCGT<br>CTTTTAATTTCAGACGCTGTTTGATCTCCCCCTTTAACGCCCCATCTTCTGGGTACATACGTTCGCTCGATGCTTCCCATCCCATAGTCTTT<br>TTCTGCATGACAGGGCCGTCTGACGGGAAATTGGTCCCCCGAAGTTTCACTTTATATATGAACTCCCCATCCTGAAGGGAGGAGTCCTGG<br>GTCACAGTGACCACTCCGCCATCTTCGAAGTTCATCACGCGCTCCCACTTGAACCCCTCGGGGAACGATAATTTAAGGTAGTCTGGTATG<br>TCGGCTGGATGCTTGACGTACGCTTTACTCCCATACATAAACTGCGGAGAAAGAATGTCCCACGCAAAAGGAAGCGGACCACCTTTAGTA<br>ACTTTTAACTTTGCCGTCTGAGTCCCCTCGTACGGGCGACCCTCGCCCTCACCCTCGATTTCAAACTCGTGGCCGTTAACGCTACCCTCC<br>ATGTGTACCTTGAATCTCATAAACTCCTTGATGATCGCCATATTGTCCTCTTCGCCCTTGCTGACCATGAATTCTTAGCCTTATTTTGGATA<br>CTATGGGTACCCTA**CCGTTTAAATTTGGCGTAGCGTTCGCGAGCAATTTCAAGGGTTGAGTTACGGACTCTTTCGAAGCGTTCTTTGTC**<br>**CTTCTGCCATAAAGAACGCACTGCAAGGCCCCGTACTGAGTTGAATATCAACCATAATATGTCCTCGGCATCATCACGTGACAGTCCC**<br>**CGCGAGACTAATACACCAAGCCACATGTCTTCAACCACGAACCGGTTACGTTCAACCGTTCTCTGAATACCTTCCCGAAGAGCTGGAT**<br>**C**ACGATCCGCGGCAACTATAAGATCAAGGGATATGGAAAAGTCATCATCAAGGAAAAATTCGGCGGCGTCGTCCAACATTTGCTGGATCA<br>CGTCATCTTCCGGCTTCAATTTAGCCAGTCTTGCCCGACTCCGTTCCGTAATCTGTTCGTACAGCCATTCGAAGGTAGCCAGCAACAGTT<br>CCAACTTAGTAGGGAAATGGTGACTCTGAGCACCCCGGCTGACTCCCGCGGCCCCAGGAACGTCAGCAATTCTGAAGCCCGCGTATCC<br>CTTCTCGCGTAAAACTCCAAGTGCAGCCGCTATCAATTTACCTTGCGTCTCCATAGCACGCTCCGCTTGGGTCCGGCGCTTAGGGGACAT<br>ATGGCAACCTCCTTGCCGCGGAGTGGAGGATCGTTGGGCGCTATCATGCCATACCGCGAAAGGTTTTGCACCATCTAGAGCGCAACGCA<br>AT**TAATGTGAGTTAGCTCACTCAT**TAGGCACCCCAGGCTTTACACTTTATGCTTCCGGCTCGTATGTTGCCTAGGAACAAACAGACAATCT<br>GGTCTGTTTGTAAACCTAAAAGAAAAAAGAGAGGAGGCATATTACTAGGACTATTAGTACCTAGTCTTAATTGTCCGGCATCCGAAACAGC<br>TAAGGAGTTACCTAGT**ATGAGCAAAGGAGAAGAACTTTTCACTGGAGTTGTCCCAATTCTTGTTGAATTAGATGGTGATGTTAATGGGC**<br>**ACAAATTTTCTGTCCGTGGAGAGGGTGAAGGTGATGCTACAAACGGAAAACTCACCCTTAAATTTATTTGCACTACTGGAAAACTACCT**<br>**GTTCCGTGGCCAACACTTGTCACTACTCTGACCTATGGTGTTCAATGCTTTTCCCGTTATCCGGATCACATGAAACGGCATGACTTTTTC**<br>**AAG**AGTGCCATGCCCGAAGGTTATGTACAGGAACGCACTATATCTTTCAAAGATGACGGGACCTACAAGACGCGTGCTGAAGTCAAGTTT<br>GAAGGTGATACCCTTGTTAATCGTATCGAGTTAAAGGGTATTGATTTTAAAGAAGATGGAAACATTCTTGGACACAAACTCGAGTACAACT<br>TTAACTCACACAATGTATACATCACGGCAGACAAACAAAAGAATGGAATCAAAGCTAACTTCAAAATTCGCCACAACGTTGAAGATGGTTC<br>CGTTCAACTAGCAGACCATTATCAACAAAATACTCCAATTGGCGATGGCCCTGTCCTTTTACCAGACAACCATTACCTGTCGACACAATCT<br>GTCCTTTCGAAAGATCCCAACGAAAAGCGTGACCACATGGTCCTTCTTGAGTTTGTAACTGCTGCTGGGATTACACATGGCATGGATGAG<br>CTCTACAAAAAGCTTGGGCCCGTTTAAACGGTCTCCAGCTTGGCTGTTTTGGCGGATGAGAGAAGATTTTCAGCCTGATACAGATTAAAT<br>CAGAACGCAGAAGCGGTCTGATAAAACAGAATTTGCCTGGCGGCAGTAGCGCGGTGGTCCCACCTGACCCCATGCCGAACTCAGAAGT<br>GAAACGCCGTAGCGCCGATGGTAGTGTGGGGTCTCCCCATGCGAGAGTAGGGAACTGCCAGGCATCAAATAAAACGAAAGGCTCAGTC<br>GAAAGACTGGGCCTTTCGTTTTATCTGTTGTTTGTCGGTGAACGCTCTCCTGAGTAGGACAAATCCGCCGGGAGCGGATTTGAACGTTGC<br>GAAGCAACGGCCCGGAGGGTGGCGGGCAGGACGCCCGCCATAAACTGCCAGGCATCAAATTAAGCAGAAGGCCATCCTGACGGATGG<br>CCTTTTTGCGTTTCTACAAACGCATGCTCTTTTTGTTTATTTTTCTAAATACATTCAAATATGTATCCGCTCATGAGACAATAACCCTGATAA<br>ATGCTTCAATAATATTGAAAAAGGAAGAGTATGAGTATTCAACATTTCCGTGTCGCCCTTATTCCCTTTTTTGCGGCATTTTGCCTTCCTGT<br>TTTTGCTCACCCAGAAACGCTGGTGAAAGTAAAAGATGCTGAAGATCAGTTGGGTGCACGAGTGGGTTACATCGAACTGGATCTCAACAG<br>CGGTAAGATCCTTGAGAGTTTTCGCCCCGAAGAACGTTTTCCAATGATGAGCACTTTTAAAGTTCTGCTATGTGGCGCGGTATTATCCCGT<br>GTTGACGCCGGGCAAGAGCAACTCGGTCGCCGCATACACTATTCTCAGAATGACTTGGTTGAGTACTCACCAGTCACAGAAAAGCATCTT<br>ACGGATGGCATGACAGTAAGAGAATTATGCAGTGCTGCCATAACCATGAGTGATAACACTGCGGCCAACTTACTTCTGACAACGATCGGA<br>GGACCGAAGGAGCTAACCGCTTTTTTGCACAACATGGGGGATCATGTAACTCGCCTTGATCGTTGGGAACCGGAGCTGAATGAAGCCAT<br>ACCAAACGACGAGCGTGACACCACGATGCCTGTAGCAATGGCAACAACGTTGCGCAAACTATTAACTGGCGAACTACTTACTCTAGCTTC<br>CCGGCAACAATTAATAGACTGGATGGAGGCGGATAAAGTTGCAGGACCACTTCTGCGCTCGGCCCTTCCGGCTGGCTGGTTTATTGCTG<br>ATAAATCTGGAGCCGGTGAGCGTGGGTCTCGCGGTATCATTGCAGCACTGGGGCCAGATGGTAAGCCCTCCCGTATCGTAGTTATCTAC<br>ACGACGGGGAGTCAGGCAACTATGGATGAACGAAATAGACAGATCGCTGAGATAGGTGCCTCACTGATTAAGCATTGGTAACTGTCAGA<br>CCAAGTTTACTCATATCCATGGATACTTTAGATTGATTTAAAACTTCATTTTTAATTTAAAAGGATCTAGGTGAAGATCCTTTTTGATAATCTC<br>ATGACCAAAATCCCTTAACGTGAGTTTTCGTTCCACTGAGCGTCAGACCCCGTAGAAAAGATCAAAGGATCTTC 3’

pCDFDuet- PhoN^{TM}-IPK-GPPS-αTOHS<br><br>PhoN^{TM} (**blue**)<br>(original sequence, GenBank: BAA11655.1)<br><br>IPK (**pink**)<br>(original sequence, NCBI: CAC11251.1)<br><br>GPPS (**orange**)<br>(original sequence, NCBI: AAN01134.1)<br><br>αTOHS (**yellow**)<br><br>(original sequence,<br>NCBI: NP_001268216.1)	GATCAAAGGATCTTCTTGAGATCCTTTTTTTCTGCGCGTAATCTTTTGCCCTGTAAACGAAAAACCACCTGGGGAGGTGGTTTGATCGAAG<br>GTTAAGTCAGTTGGGGAACTGCTTAACCGTGGTAACTGGCTTTCGCAGAGCACAGCAACCAAATCTGTCCTTCCAGTGTAGCCGGACTTT<br>GGCGCACACTTCAAGAGCAACCGCGTGTTTAGCTAAACAAATCCTCTGCGAACTCCCAGTTACCAATGGCTGCTGCCAGTGGCGTTTTAC<br>CGTGCTTTTCCGGGTTGGACTCAAGTGAACAGTTACCGGATAAGGCGCAGCAGTCGGGCTGAACGGGGAGTTCTTGCTTACAGCCCAGC<br>TTGGAGCGAACGACCTACACCGAGCCGAGATACCAGTGTGTGAGCTATGAGAAAGCGCCACACTTCCCGTAAGGGAGAAAGGCGGAAC<br>AGGTATCCGGTAAACGGCAGGGTCGGAACAGGAGAGCGCAAGAGGGAGCGACCCGCCGGAAACGGTGGGGATCTTTAAGTCCTGTCG<br>GGTTTCGCCCGTACTGTCAGATTCATGGTTGAGCCTCACGGCTCCCACAGATGCACCGGAAAAGCGTCTGTTTATGTGAACTCTGGCAG<br>GAGGGCGGAGCCTATGGAAAAACGCCACCGGCGCGGCCCTGCTGTTTTGCCTCACATGTTAGTCCCCTGCTTATCCACGGAATCTGTGG<br>GTAACTTTGTATGTGTCCGCAGCGCTTTTCTACTGAACCGCTCTAGATTTCAGTGCAATTTATCTCTTCAAATGTAGCACCTGAAGTCAGC<br>CCCATACGATATAAGTTGTAATTCTCATGTTAGTCATGCCCCGCGCCCACCGGAAGGAGCTGACTGGGTTGAAGGCTCTCAAGGGCATC<br>GGTCGAGATCCCGGTGCCTAATGAGTGAGCTAACTTACATTAATTGCGTTGCGCTCACTGCCCGCTTTCCAGTCGGGAAACCTGTCGTG<br>CCAGCTGCATTAATGAATCGGCCAACGCGCGGGGAGAGGCGGTTTGCGTATTGGGCGCCAGGGTGGTTTTTCTTTTCACCAGTGAGACG<br>GGCAACAGCTGATTGCCCTTCACCGCCTGGCCCTGAGAGAGTTGCAGCAAGCGGTCCACGCTGGTTTGCCCCAGCAGGCGAAAATCCT<br>GTTTGATGGTGGTTAACGGCGGGATATAACATGAGCTGTCTTCGGTATCGTCGTATCCCACTACCGAGATGTCCGCACCAACGCGCAGC<br>CCGGACTCGGTAATGGCGCGCATTGCGCCCAGCGCCATCTGATCGTTGGCAACCAGCATCGCAGTGGGAACGATGCCCTCATTCAGCA<br>TTTGCATGGTTTGTTGAAAACCGGACATGGCACTCCAGTCGCCTTCCCGTTCCGCTATCGGCTGAATTTGATTGCGAGTGAGATATTTATG<br>CCAGCCAGCCAGACGCAGACGCGCCGAGACAGAACTTAATGGGCCCGCTAACAGCGCGATTTGCTGGTGACCCAATGCGACCAGATGC<br>TCCACGCCCAGTCGCGTACCGTCTTCATGGGAGAAAATAATACTGTTGATGGGTGTCTGGTCAGAGACATCAAGAAATAACGCCGGAACA<br>TTAGTGCAGGCAGCTTCCACAGCAATGGCATCCTGGTCATCCAGCGGATAGTTAATGATCAGCCCACTGACGCGTTGCGCGAGAAGATT<br>GTGCACCGCCGCTTTACAGGCTTCGACGCCGCTTCGTTCTACCATCGACACCACCACGCTGGCACCCAGTTGATCGGCGCGAGATTTAA<br>TCGCCGCGACAATTTGCGACGGCGCGTGCAGGGCCAGACTGGAGGTGGCAACGCCAATCAGCAACGACTGTTTGCCCGCCAGTTGTTG<br>TGCCACGCGGTTGGGAATGTAATTCAGCTCCGCCATCGCCGCTTCCACTTTTTCCCGCGTTTTCGCAGAAACGTGGCTGGCCTGGTTCA<br>CCACGCGGGAAACGGTCTGATAAGAGACACCGGCATACTCTGCGACATCGTATAACGTTACTGGTTTCACATTCACCACCCTGAATTGAC<br>TCTCTTCCGGGCGCTATCATGCCATACCGCGAAAGGTTTTGCGCCATTCGATGGTGTCCGGGATCTGCGAAATTAATACGACTCACTATA<br>GGGGAATTGTGAGCGGATAACAATTCCCCTCTAGAAATAATTTTGTTTAACTTTAAGAAGGAGATATACCATGGGCAGCAGCCATCACCAT<br>CATCACCACAGCCAGGATCCG**ATGAAACGTCAGCTGTTTACCCTGAGCATCGTGGGCGTGTTTAGCCTGAACACCTTTGCCAGCATTC**<br>**CGCCGGGTAATGACGTGACCACCAAGCCGGACCTGTATTACCTGACCAACGATAACGCCATCGACAGCCTGGCACTGTTACCGCCGC**<br>**CTCCACAGATTGGCAGCATCGCCTTTCTGAATGACCAGGCAATGTATGAGAAGGGCCGCCTGCTGCGTAATACCGAACGCGGCAAGC**<br>**TGGCCGCAGAAGAT**GCCAATCTGAGCAGTGGCGGCGTGGCCAATGTTTTCAGTGCCGCCTTTGGCAGCCCGATTACCGCCAAAGATAG<br>CCCGGAACTGCACAAGCTGCTGACCAACATGATCAGAGACGCCGGTGATCTGGCCACCCGTAGCGCCAAAGAATACTACATGCGTATTC<br>GCCCGTTCGCCTTCTATGGCGTGAGCACCTGTAACACCAAGGAACAGGATAAGCTGAGCAAAAACGGCAGCTATCCGAGCGGTCATACC<br>AGCATTGGTTGGGCCACCGCACTGGTGCTGAGCGAAATCAATCCGGCCCGCCAGGATACCATTCTGAAACGCGGCTACGAACTGGGTG<br>ATAGCCGCGTGATTTGCGGCTACCATTGGCAAAGCGATGTGGATGCAGCCCGTATTGTTGGCAGCGCCATCGTGGCAACCCTGCATAGC<br>AATCCGGTGTTTCAGGCACAGCTGCAGAAAGCCAAGGATGAATTCGCCAATAATCAGAAAAAATAAAGATTGCGATAAACAATAACGTTCC<br>CGGAATTGATGGAGATTGCTAGTTAAGTATAAGAAGGAGATATACATATGATAGATCCGTTCACC**ATGATGATCCTGAAGATTGGCGGCA**<br>**GCGTGATTACCGATAAGAGCGCATATCGCACCGCACGCACCTACGCCATTCGTAGCATCGTGAAAGTGCTGAGCGGCATTGAAGATC**<br>**TGGTGTGCGTGGTGCATGGCGGTGGTAGCTTTGGCCACATCAAGGCGATGGAGTTTGGTCTGCCGGGTCCGAAAAATCCGCGTAGCA**<br>**GCATCGGCTACAGCATCGTGCATCGCGACATGGAAAACCTGGACCTGATGGTGATC**GACGCAATGATCGAGATGGGTATGCGCCCGA<br>TTAGCGTGCCGATTAGCGCCCTGCGTTATGATGGTCGCTTTGACTACACCCCGCTGATCCGCTATATTGATGCAGGCTTCGTGCCGGTG<br>AGCTATGGCGACGTGTATATCAAGGACGAACATAGCTATGGCATCTACAGCGGCGACGATATTATGGCCGATATGGCCGAACTGCTGAA<br>GCCGGATGTGGCCGTGTTCCTGACCGATGTGGATGGCATCTATAGCAAGGACCCGAAACGCAATCCGGATGCCGTGCTGCTGCGCGAC<br>ATCGATACAAACATCACCTTCGATCGCGTGCAGAACGATGTGACCGGCGGCATTGGCAAGAAATTCGAAAGCATGGTTAAAATGAAAAGT<br>AGCGTTAAAAATGGTGTGTACCTGATTAATGGCAATCACCCGGAGCGCATTGGTGACATCGGCAAGGAGAGCTTCATCGGTACCGTGATT<br>CGCTAATGTCAGCCTTTCCGATTAATACGACTCACTATAGGGGAATTGTGAGCGGATAACAATTCCCCTGTAGAAATAATTTTGTTTAACTT<br>TAATAAGGAGATATAATGGAATTTGACTTCAACAAATAC**ATGGACTCCAAAGCGATGACGGTAAATGAAGCACTGAACAAAGCGATCCC**<br>**TCTGCGTTATCCGCAGAAAATCTACGAAAGCATGCGTTACAGCCTGCTGGCAGGCGGCAAGCGTGTTCGTCCGGTTCTGTGTATTGCC**<br>**GCATGTGAACTGGTAGGTGGTACCGAAGAACTGGCGATCCCGACCGCGTGCGCAATTGAAATGATCCACACGATGTCCCTGATGCAC**<br>**GATGATCTGCCGTGTATCGACAACGACGAT**CTGCGTCGCGGTAAACCGACTAACCACAAAATTTTCGGTGAGGATACCGCAGTGACTGC<br>TGGTAACGCACTGCACTCTTACGCCTTCGAGCATATCGCGGTTTCTACTTCTAAAACCGTTGGTGCTGACCGCATCCTGCGTATGGTGTC<br>CGAGCTGGGTCGTGCTACTGGCTCTGAAGGTGTTATGGGTGGTCAGATGGTAGACATCGCATCCGAAGGCGATCCGTCTATCGACCTGC<br>AGACCCTGGAATGGATTCACATCCACAAAACCGCAATGCTGCTGGAATGCTCCGTTGTTTGCGGTGCAATCATTGGCGGTGCCAGCGAA<br>ATCGTAATCGAACGTGCCCGTCGCTACGCCCGCTGTGTTGGTCTGCTGTTCCAGGTAGTTGATGACATTCTGGACGTAACTAAAAGCAGC<br>GACGAACTGGGTAAGACTGCGGGCAAGGACCTGATCTCTGATAAAGCCACCTACCCAAAGCTGATGGGTCTGGAAAAGGCCAAGGAGTT<br>CTCCGATGAACTGCTGAACCGTGCGAAGGGTGAACTGTCCTGCTTCGACCCAGTTAAAGCCGCTCCGCTGCTGGGCCTGGCAGACTAC<br>GTGGCATTTCGTCAGAATTAATCGAACAGAAAGTAATCGTATTGTACACGGCCGCATAATCGAAATTAATACGACTCACTATAGGGGAATT<br>GTGAGCGGATAACAATTCCCCATCTTAGTATATTAGTTAAGTATAAGAAGGAGTATACAT**ATGGCTTCGCCTCGTGGTATAAAAGTCAAAA**<br>**TAGGGAATTCTAATTGTGAAGAAATCATTGTGCGTCGCACAGCAAACTACCATCCCACCATTTGGGACTACGACTATGTCCAGTCCTTG**<br>**CGTTCAGATTATGTTGGAGAGACTTACACGAGACGCCTTGACAAGCTTAAGCGGGATGTGAAACCCATGTTAGGTAAGGTAAAGAAG**

	**CCCCTTGACCAGTTAGAACTGATAGACGTTTTACAAAGATTAGGGATT**TACTATCATTTCAAGGACGAGATAAAACGGATTTTGAACGGC<br>ATTTACAATCAATACAATAGACACGAAGAGTGGCAAAAGGATGATTTATATGCCACTGCACTGGAGTTCAGATTGCTGCGGCAGCACGGG<br>TACGATGTTCCACAGGATGTCTTTTCACGCTTTAAAGATGACACCGGCTCTTTCAAGGCATGTTTGTGTGAGGATATGAAAGGCATGCTTT<br>GCCTTTACGAAGCTAGTTACTTGTGTGTCCAAGGCGAGTCTACGATGGAGCAAGCGCGGGACTTTGCGCACCGGCACTTGGGCAAAGG<br>CCTGGAACAAAACATAGACCAGAACTTGGCCATAGAAGTTAAACATGCTTTGGAGTTGCCGCTGCATTGGCGGATGCCTAGATTGGAGG<br>CACGGTGGTTTATAGACGTGTATGAAAAACGTCAAGACATGAATCCCATCCTGCTTGAGTTCGCTAAACTTGATTTTAACATGGTGCAGGC<br>GACGCATCAGGAGGACCTTAGACATATGTCGTCATGGTGGTCCTCTACACGCTTAGGAGAAAAATTAAATTTCGCACGGGATCGTTTAAT<br>GGAGAACTTCTTGTGGACTGTCGGCGTGATTTTCGAGCCGCAATATGGTTATTGCCGTCGTATGAGCACTAAAGTGAATACGTTAATAAC<br>CATTATAGACGACGTTTATGACGTATACGGAACTATGGACGAATTAGAATTATTCACAGACGTTGTGGACCGCTGGGACATTAACGCCAT<br>GGACCCTCTTCCTGAATACATGAAACTTTGCTTTCTTGCCCTTTACAATAGCACAAACGAGATGGCATACGACGCTCTTAAGGAACATGGT<br>CTGCACATCGTATCCTATTTACGGAAAGCCTGGTCGGACCTTTGCAAGAGCTATTTGTTAGAGGCAAAATGGTACTACAGTAGATATACTC<br>CGAGTTTACAAGAGTATATTAGTAACAGTTGGATCAGCATCTCAGGTCCGGTGATCTTAGTCCACGCGTACTTTCTGGTAGCTAATCCGAT<br>CACTAAAGAGGCCTTGCAATCATTGGAACGCTATCACAATATCATCAGATGGTCTTCAATGATTTTAAGATTGAGTGACGACCTGGGAACT<br>TCATTAGACGAACTGAAACGCGGTGATGTCCCCAAGTCTATTCAGTGCTATATGTACGAAACCGGAGCGTCCGAAGAAGATGCTAGAAAG<br>CACACCAGCTATTTGATTGGGGAAACATGGAAGAAGTTGAACGAGGACGGTGCTGTAGAGAGTCCATTTCCTGAAACGTTCATTGGGATA<br>GCTATGAATCTTGCTCGGATGGCTCAGTGTATGTATCAACACGGAGACGGGCACGGTATCGAATACGGGGAAACAGAAGACCGGGTACT<br>GTCATTGTTAGTGGAGCCCATCCCGTCGCTGTCTTCTGAGTAGCTGCCACCGCTGAGCAATAACTAGCATAACCCCTTGGGGCCTCTAAA<br>CGGGTCTTGAGGGGTTTTTTGCTGAAACCTCAGGCATTTGAGAAGCACACGGTCACACTGCTTCCGGTAGTCAATAAACCGGTAAACCAG<br>CAATAGACATAAGCGGCTATTTAACGACCCTGCCCTGAACCGACGACCGGGTCATCGTGGCCGGATCTTGCGGCCCCTCGGCTTGAACG<br>AATTGTTAGACATTATTTGCCGACTACCTTGGTGATCTCGCCTTTCACGTAGTGGACAAATTCTTCCAACTGATCTGCGCGCGAGGCCAAG<br>CGATCTTCTTCTTGTCCAAGATAAGCCTGTCTAGCTTCAAGTATGACGGGCTGATACTGGGCCGGCAGGCGCTCCATTGCCCAGTCGGC<br>AGCGACATCCTTCGGCGCGATTTTGCCGGTTACTGCGCTGTACCAAATGCGGGACAACGTAAGCACTACATTTCGCTCATCGCCAGCCC<br>AGTCGGGCGGCGAGTTCCATAGCGTTAAGGTTTCATTTAGCGCCTCAAATAGATCCTGTTCAGGAACCGGATCAAAGAGTTCCTCCGCC<br>GCTGGACCTACCAAGGCAACGCTATGTTCTCTTGCTTTTGTCAGCAAGATAGCCAGATCAATGTCGATCGTGGCTGGCTCGAAGATACCT<br>GCAAGAATGTCATTGCGCTGCCATTCTCCAAATTGCAGTTCGCGCTTAGCTGGATAACGCCACGGAATGATGTCGTCGTGCACAACAATG<br>GTGACTTCTACAGCGCGGAGAATCTCGCTCTCTCCAGGGGAAGCCGAAGTTTCCAAAAGGTCGTTGATCAAAGCTCGCCGCGTTGTTTC<br>ATCAAGCCTTACGGTCACCGTAACCAGCAAATCAATATCACTGTGTGGCTTCAGGCCGCCATCCACTGCGGAGCCGTACAAATGTACGG<br>CCAGCAACGTCGGTTCGAGATGGCGCTCGATGACGCCAACTACCTCTGATAGTTGAGTCGATACTTCGGCGATCACCGCTTCCCTCATA<br>CTCTTCCTTTTTCAATATTATTGAAGCATTTATCAGGGTTATTGTCTCATGAGCGGATACATATTTGAATGTATTTAGAAAAATAAACAAATA<br>GCTAGCTCACTCGGTCGCTACGCTCCGGGCGTGAGACTGCGGCGG 3’

Supplementary Table S2: Oligonucleotides (5'—3') used in this study. Mutations are in lower case.

Primer #	Name	Nucleotide Sequence	Function
Construction of pSENSE-CymR biosensor			
1	cuO-F	GTTTCCTCCTGTTAGCagaaacaaaccaacctgtctgtattaTTATATTCAATCCCACAC	
2	cuO-R	GTGTGGGATTGAATATAAtaatacagacaggttggtttgtttctGCTAACAGGAGGAAAC	cloning the operator sequence (cuO)
3	cym-F	ataCATATGTCCCCTAAGC	
4	cym-R	ataGGTACCCTACCG	amplification of *CymR*
Site-directed mutagenesis of CymR variants			
5	107A-F	GGGATATGGAgcaGTCATCATCAAG	
6	107A-R	TTGATCTTATAGTTGCCG	alanine at Phe107
7	168L-F	CTGCAAGGCCctcTACTGAGTTGAATATC	
8	168L-R	TGCGTTCTTTATGGCAGAAG	leucine at Arg168
9	172A-F	GCCATAAAGAACGCgaTGCAAGGCCCC	
10	172A-R	GATATTCAACTCAGTACGGGGCCTTGC	alanine at Val172
11	168L/172A-F	GGGATATGGAgcaGTCATCATCAAG	
12	168L/172A-R	TTGATCTTATAGTTGCCG	leucine and alanine at Arg168 and Val172
Saturation mutagenesis of CymR			
13	R168X/V172X-F	GTTGATATTCAACTCAGTANNKGGCCTTGCANNKCGTTCTTTATG	
14	R168X/V172X-R	CATAATATGTCCTCGGCATCATCAC	saturation library of Arg168 and Val172
15	F107X-F	CGCCGAATTTTTCCTTGATGATGACNNKTCCATATCCC	
16	F107X-R	GCGTCGTCCAACATTTGCTGG	saturation library of Phe107
Construction of αTOH production plasmids			
17	pCDFBB-F	TCGGTACCGTGATTCGCTAAATTAATACGACTCACTATAGGGGA	
18	pCDFBB-R	CCCTATAGTGAGTCGTATTAGATCCCGGACACCATCGAAT	linearizing pCDFDuet for ADH
19	ADH-F	ATTCGATGGTGTCCGGGATCTAATACGACTCACTATAGGGGA	
20	ADH-R	CTATAGTGAGTCGTATTAATTTAGCGAATCACGGTACCGA	amplification of *PhoN/lPK* fragment
21	GPPs-F	AACTTTAATAAGGAGATATAATGGAATTTGACTTCAACAAATACATGG	
22	GPPs-R	TACGATTACTTTCTGTTCGATTAATTCTGACGAAATGCCAC	amplification of *GPPs*
23	αTOHs-F	AAGTATAAGAAGGAGATATACTCAGAAGACAGCGACGGGA	
24	αTOHs-R	TTGCTCAGCGGTGGCAGCAGATGGCTCTTAGCATGTTGTCT	amplification of *αTOHs*
25	pCDFBBv2-F	TGGCATTTCGTCAGAATTAATCGAACAGAAAGTAATCGTATTG	
26	pCDFBBv2-R	TTTGTTGAAGTCAAATTCCATTATATCTCCTTATTAAAGTTAAACAAAATTATTTC	linearizing pCDFDuet for *GPPs*

27	pCDFBBv3-F	GACAACATGCTAAGAGCCATCTGCTGCCACCGCTGAGCAA	
28	pCDFBBv3-R	TCCCGTCGCTGTCTTCTGAGTATATCTCCTTCTTATACTTAACTAATATACTAAGATG	linearizing pCDFDuet for *αTOHs*
29	RBS_PhON-R	ATTCGATGGTGTCCGGGATCTGCGAAATTAATACGACTCAC	
30	RBS_PhON-R	CTATAGTGAGTCGTATTAATCGGATCTTTATTTTTTCTGATTATTGG	amplification of RBS-PhoN fragment
31	pCDFBB_αTOHs-F	CGCCAATAATCAGAAAAAATAATAATCGAACAGAAAGTAATCGTAT	
32	pCDFBB_αTOHs-R	GTAAACAGCTGACGTTTCATTATATCTCCTTATTAAAGTTAAACAAAATTATTT	linearizing pCDFDuet-αTOHs for *GPPs*
33	RBS-PhoNv2-F	AACTTTAATAAGGAGATATAATGAAACGTCAGCTGTTTACC	
34	RBS_PhoNv2-R	GATTACTTTCTGTTCGATTATTATTTTTTCTGATTATTGGCGAAT	amplification of RBS-PhoN fragment
35	pCDFBB_GPPs/αTOHs-F	TCAGAAAAAATAAAGATCCGATTAATACGACTCACTATAGGGGA	
36	pCDFBB_GPPs/αTOHs-R	TGAGTCGTATTAATTTCGCAGATCCCGGACACCATCGAAT	
37	IPK-F	CCGGAATTGATGGAGATTGCTAGTTAAGTATAAGAAGGAGATATACATATG	
38	IPK-R	ATTAATCGGAaaggcTGACATTAGCGAATCACGGTACCGA	amplification of *IPK*
39	pCDFBB_PhoN/GPPs/αTOHs-F	TCGGTACCGTGATTCGCTAATGTCAgcctttTCCGATTAAT	
40	pCDFBB_PhoN/GPPs/αTOHs-R	CTCCTTCTTATACTTAACTAGCAATCTCCATCAATTCCGG	linearizing pCDFDuet-PhoN/GPPs/αTOHs for *IPK*
Site-directed mutagenesis of PhoN variants			
41	157K/160K-F	agcaaaAACGGCAGCTATCCGAGC	
42	157K/160K-R	cagcttATCCTGTTCCTTGGTGTTACAG	lysine at Thr157 and Arg160
43	122R-F	GTCAGCAGCTTGTGCAGT	
44	122R-R	CAACATGATCagaGACGCCGGTG	arginine at Glu122

Supplementary Figures

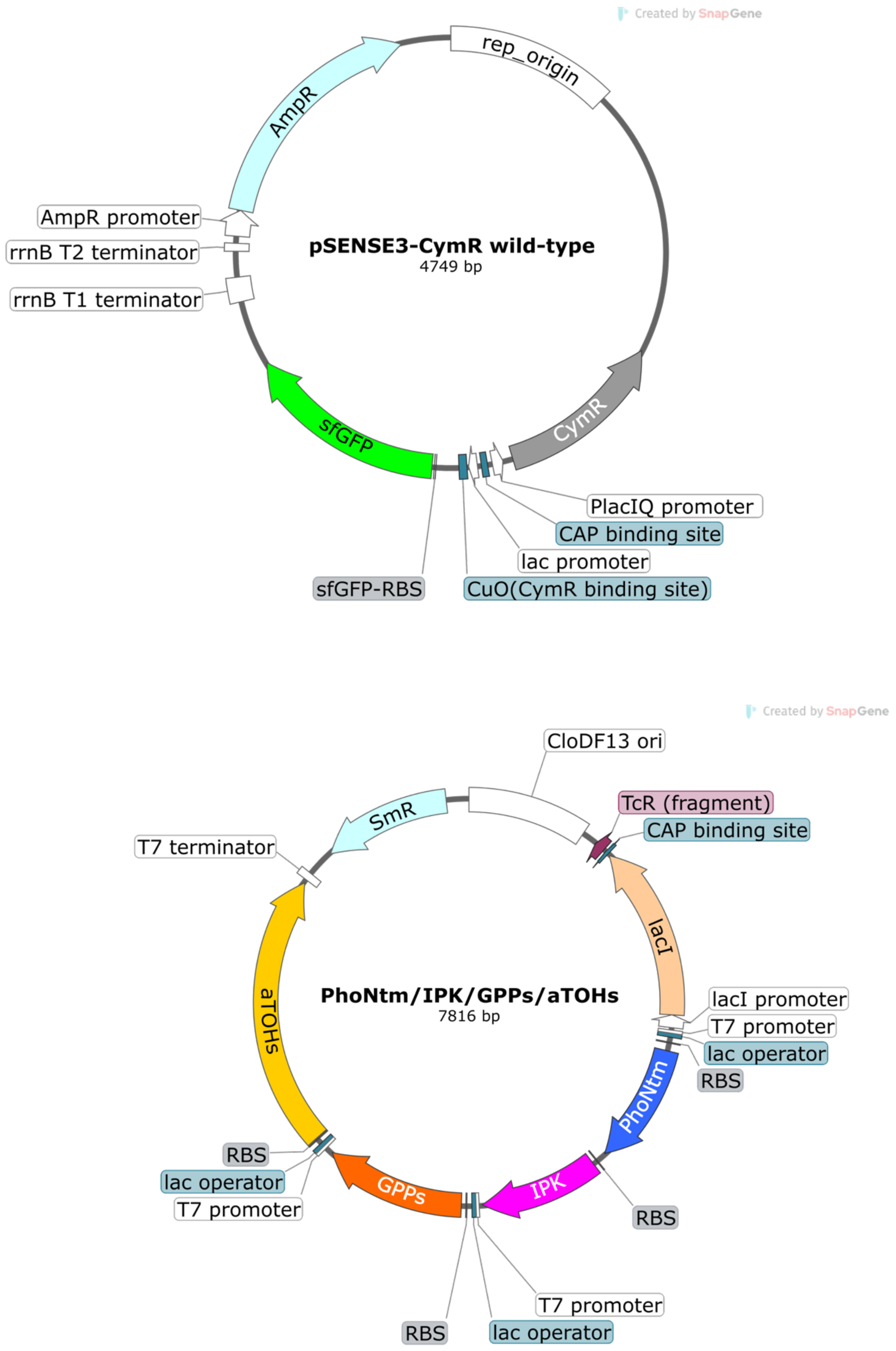

Supplementary Figure S1. The plasmid map of pSENSE3-CymR-*sf*GFP (top) and pCDFDuet-PhoN-IPK-GPPs-αTOHS (bottom).

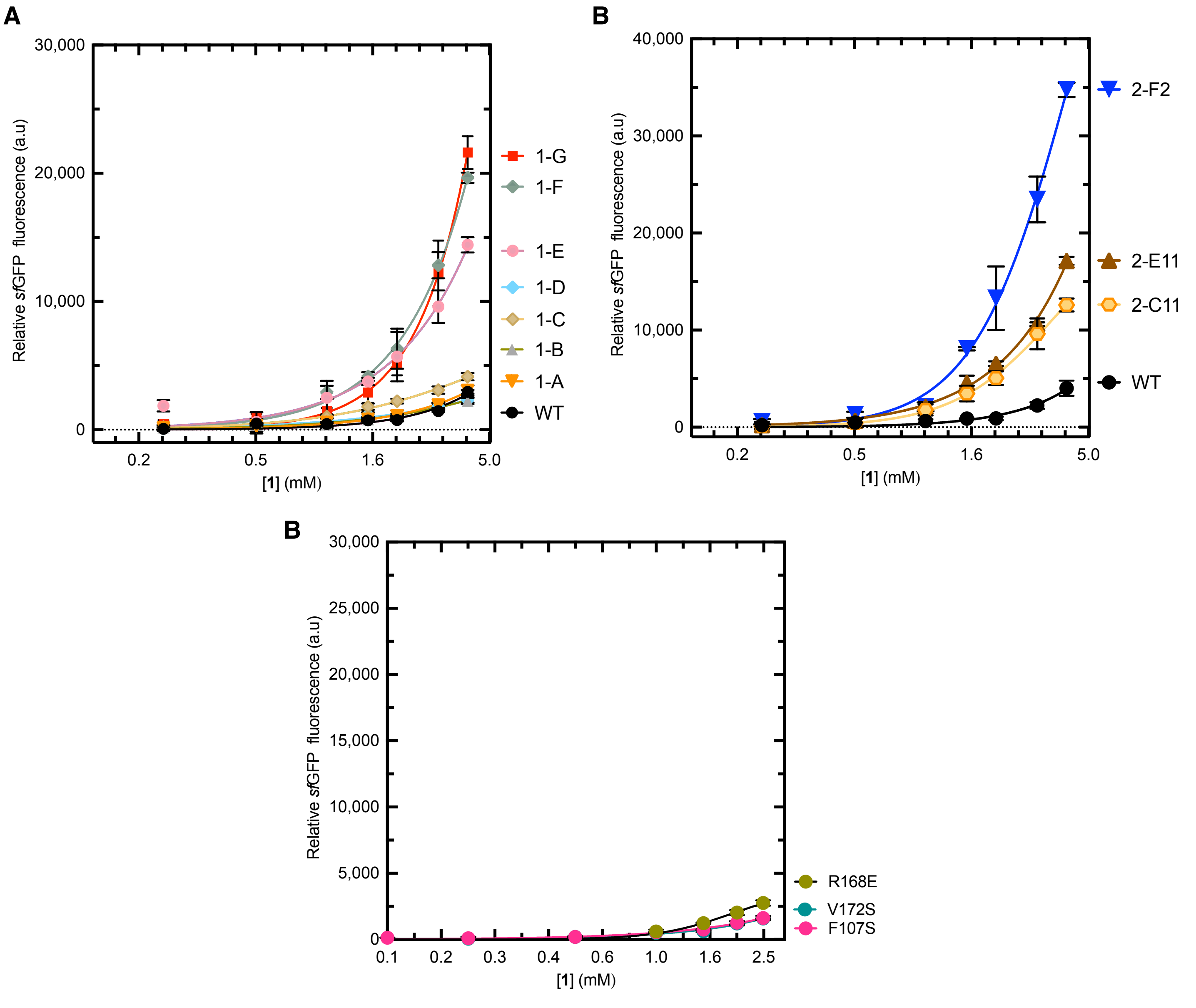

Supplementary Figure S2. Performance of wild-type and CymR variants with **1**. Dose-response curves for the wild-type and variant biosensor strains obtained via **A**) site-directed mutagenesis at 4.0 mM **1**; **B**) second-generation mutants obtained via multisite saturation library; **C**) single mutant site-directed mutagenesis derivatives of 3-A8.

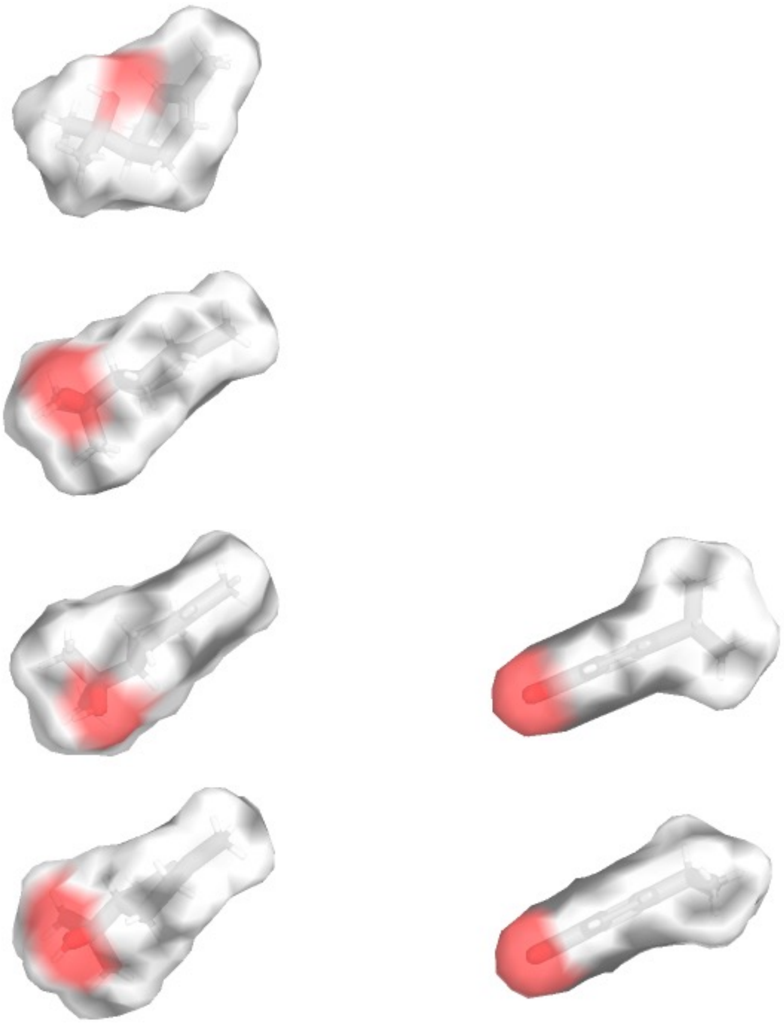

α-terpineol (1)
MW = 154.25 g/mol
XLogP3-AA = 1.8
Volume = 132.2 Å^3
Topological Polar Surface Area = 20.2 Å^2

***p*-cumate**
MW = 163.19 g/mol
XLogP3-AA = 4
Volume = 135.6 Å^3
Topological Polar Surface Area = 40.1 Å^2

Supplementary Figure S3. 3D structures of **1** and *p*-cumate. Structures are displayed as ball-and-stick models with their electrostatic surfaces. Conformations of each compound, as reported in PubChem (PubChem CID 17100 and 3417835, respectively), were viewed in PyMol.

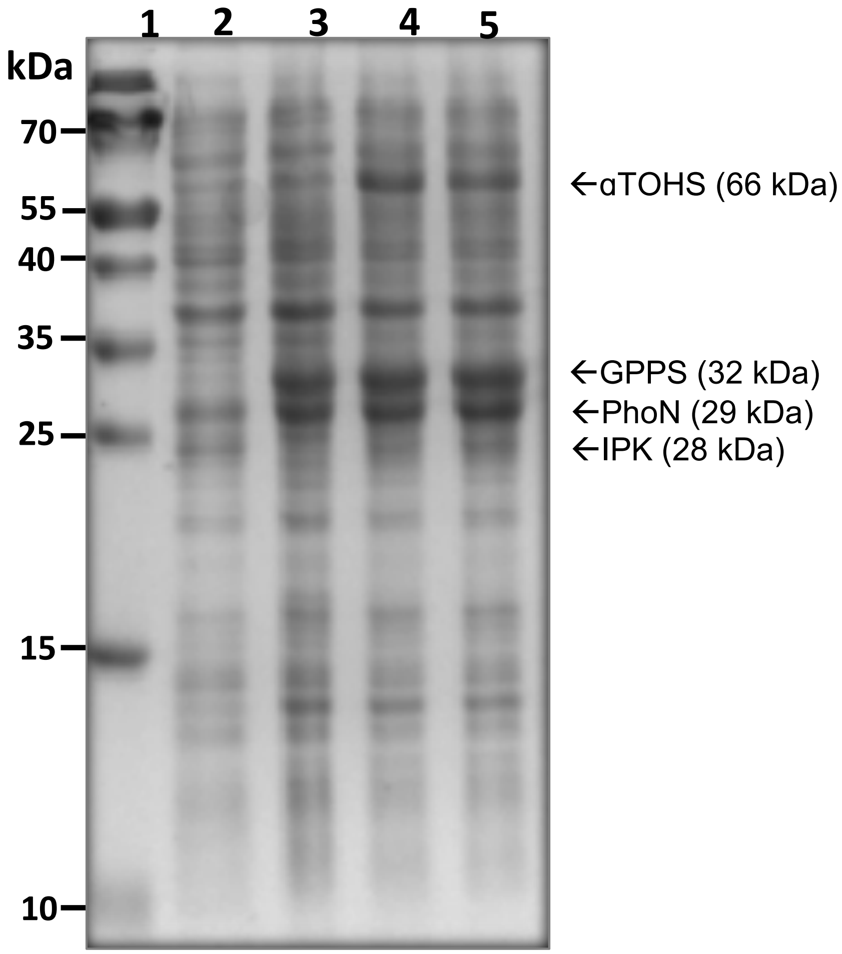

Supplementary Figure S4. SDS-PAGE analysis of **1-**production and deletion strains. Lane 1, protein marker; Lane 2, whole cell lysate of *E. coli* BL21(DE3) pCDFDuet-PhoN$_{Sf}$^{TM}-IPK$_{Ta}$; Lane 3, *E. coli* BL21(DE3) pCDFDuet-PhoN$_{Sf}$^{TM}-GPPS$_{Ag}$; Lane 4, *E. coli* BL21(DE3) pCDFDuet-PhoN$_{Sf}$^{TM}-GPPS$_{Ag}$-αTOHS$_{Vv}$; and Lane 5, *E. coli* BL21(DE3) pCDFDuet-PhoN$_{Sf}$^{TM}-IPK$_{Ta}$-GPPS$_{Ag}$-αTOHS$_{Vv}$. The expected MW based on the protein sequence is shown in brackets.

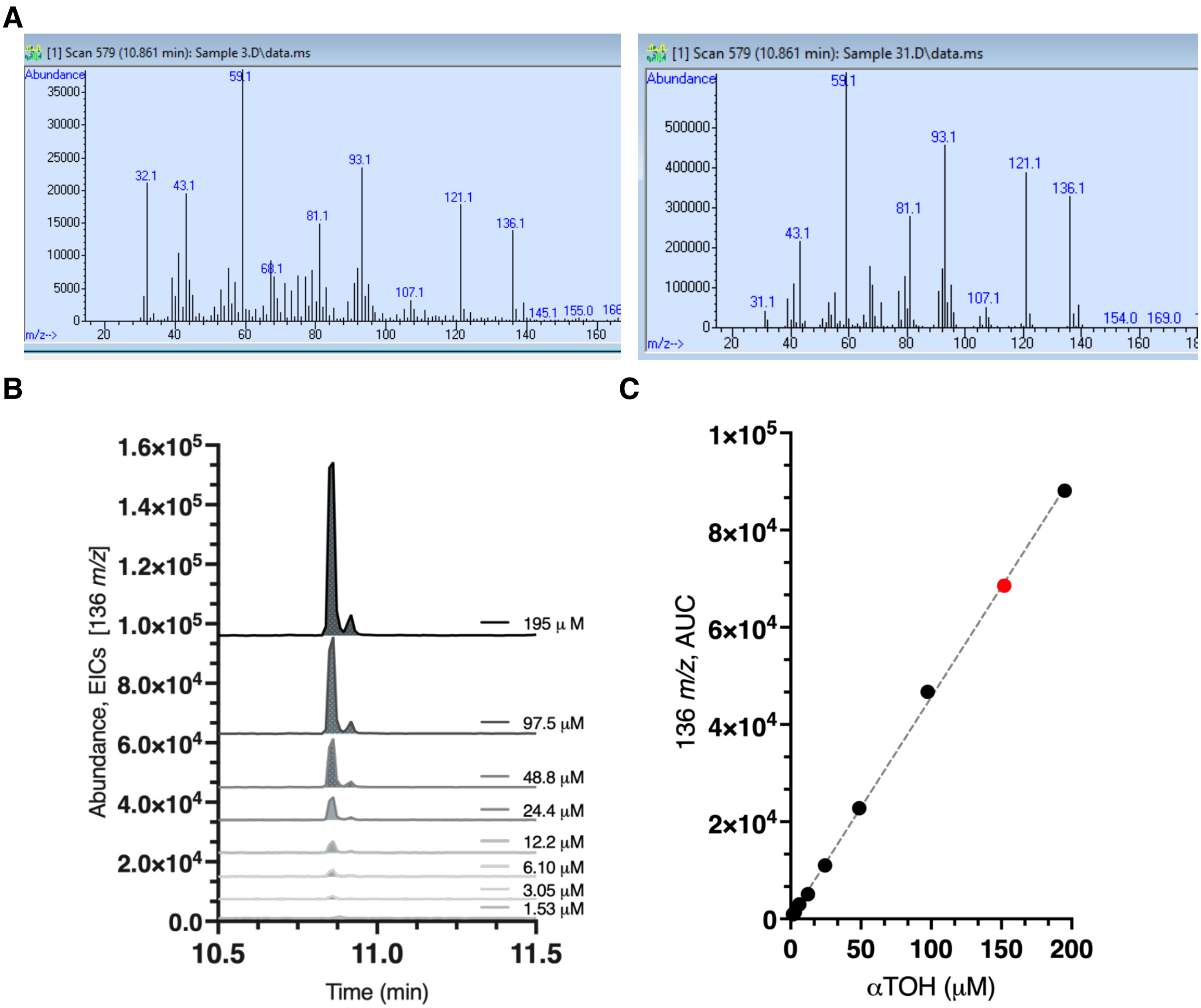

Supplementary Figure S5. GC–MS identification and quantification of **1** from culture extracts. **A**) Left: Mass spectrum of strain produced **1** showing characteristic fragment ions at *m/z* 59, 93, 121, and 136, confirming compound identity. Right: Mass spectrum of commercial standard **1**. **B**) Extracted ion chromatograms (EIC, *m/z* = 136) of **1** via GC-MS analysis. **C**) Calibration curve of **1** using EIC peak areas. The full pCDFDuet-PhoN^{TM}-IPK-GPPS-αTOHS pathway (red) produced an estimated **1** concentration of ~150 µM in culture.

Supplementary References

1. Kalkreuter, E., et al., *Development of a Genetically Encoded Biosensor for Detection of Polyketide Synthase Extender Units in Escherichia coli.* ACS SYNTHETIC BIOLOGY, 2019. **8**(6): p. 1391-1400.

2. Gayen, A., et al., *Promiscuity of an Alcohol-Dependent Hemiterpene Pathway for the In Vivo Production of a Non-Natural Alkylated Tryptophan Derivative.* ACS SYNTHETIC BIOLOGY, 2025. **14**(4): p. 1220-1229.
